# Adverse Effects of Antidepressants for Chronic Pain: A Systematic Review and Meta-analysis

**DOI:** 10.3389/fneur.2017.00307

**Published:** 2017-07-14

**Authors:** Carina Riediger, Tibor Schuster, Kristian Barlinn, Sarah Maier, Jürgen Weitz, Timo Siepmann

**Affiliations:** ^1^Department of General, Thoracic and Vascular Surgery, University Hospital Carl Gustav Carus, Technische Universität Dresden, Dresden, Germany; ^2^Center for Clinical Research and Management Education, Division of Health Care Sciences, Dresden International University, Dresden, Germany; ^3^Department of Family Medicine, McGill University, Montreal, QC, Canada; ^4^Department of Neurology, Carl Gustav Carus University Hospital, Technische Universität Dresden, Dresden, Germany; ^5^Department of Pedriatric Oncology, University Hospital Eppendorf, Universität Hamburg, Hamburg, Germany

**Keywords:** antidepressants, side effects, safety, chronic pain, amitriptyline, nortriptyline, venlafaxine, fluoxetine

## Abstract

**Background:**

Antidepressants are widely used in the treatment of chronic pain. Applied doses are lower than those needed to unfold an antidepressive effect. While efficacy of antidepressants for chronic pain has been reported in large randomized-controlled trials (RCT), there is inconsistent data on adverse effects and tolerability. We aimed at synthesizing data from RCT to explore adverse effect profiles and tolerability of antidepressants for treatment of chronic pain.

**Methods:**

Systematic literature research and meta-analyses were performed regarding side effects and safety of different antidepressants in the treatment of chronic pain according to Preferred Reporting Items for Systematic Reviews and Meta-Analyses guidelines. The National Center for Biotechnology Information library and MEDLINE were searched. Randomized placebo-controlled trials were included in quantitative data synthesis.

**Results:**

Out of 1,975 screened articles, 33 papers published between 1995 and 2015 were included in our review and 23 studies were included in the meta-analyses. A higher risk for adverse effects compared to placebo was observed in all antidepressants included in our analyses, except nortriptyline. The most prevalent adverse effects were dry mouth, dizziness, nausea, headache, and constipation. Amitriptyline, mirtazapine, desipramine, venlafaxine, fluoxetine, and nortriptyline showed the highest placebo effect-adjusted risk of adverse effects. Risk for withdrawal due to adverse effects was highest in desipramine (risk ratio: 4.09, 95%-confidence interval [1.31; 12.82]) followed by milnacipran, venlafaxine, and duloxetine. The most common adverse effects under treatment with antidepressants were dry mouth, dizziness, nausea, headache, and constipation followed by palpitations, sweating, and drowsiness. However, overall tolerability was high. Each antidepressant showed distinct risk profiles of adverse effects.

**Conclusion:**

Our synthesized data analysis confirmed overall tolerability of low-dose antidepressants for the treatment of chronic pain and revealed drug specific risk profiles. This encompassing characterization of adverse effect profiles might be useful in defining multimodal treatment regimens for chronic pain which also consider patients’ comorbidities and co-medication.

## Introduction

Chronic pain is a prevalent condition which affects 36% of the population in US (19% in Europe, respectively) and reduces quality of life. Moreover, it constitutes a considerable socioeconomic burden due to health-care resource consumption with annual costs of up to $43 billion per year (1, 2). Treatment of chronic pain is challenging since etiologies are heterogeneous, including *inter alia* diabetic neuropathy, osteoarthritis, fibromyalgia, and headache syndromes such as migraine (3).

While multimodal treatment regimens including both pharmacological and non-pharmacological interventions are most effective in the treatment of chronic pain, pain medication is still the second most prescribed group of drugs in the US alone, accounting for 12% of all prescriptions (2). Traditional agents, such as opioids and non-steroidal anti-inflammatory drugs (NSAIDs), are efficacious in the treatment of chronic pain but they are limited by adverse effects, tolerance, and potential for addiction. Although not specifically intended to treat chronic pain, various antidepressants were shown in large randomized-controlled trials (RCT) to be efficacious in the treatment of chronic pain conditions, such as diabetic neuropathy or migraine (1). Notably, required dosages to achieve an analgesic effect are lower than those needed to unfold an antidepressive effect. However, there is inconsistent data on adverse effects and tolerability of antidepressants in the treatment of chronic pain. This is clinically relevant since patients with chronic pain are frequently treated with multiple drugs, leading to increased risk of drug interactions and additive adverse effects (4, 5). Additionally, neither analgesic mechanisms of action of antidepressants nor pathophysiology of chronic pain are fully elucidated, highlighting the necessity of improving our knowledge on clinical adverse effects of these drugs (6, 7).

Adverse effect profiles of antidepressants may differ based on their specific pharmacodynamic and pharmacokinetic characteristics, an overview of which is given in Tables S1 and S2 in Supplementary Material.

Meta-analyses of efficacy and safety exist for specific antidepressants. We aimed to undertake an encompassing synthesized analysis of adverse effects of the most widely used antidepressants in the treatment of chronic pain. In particular, we sought to evaluate tolerability and risk of adverse effects related to antidepressants in the therapy of chronic pain.

## Materials and Methods

### Literature Search Strategy

We performed a systematic review and meta-analysis according to the Preferred Reporting Items for Systematic Reviews and Meta-Analyses guidelines. We systematically searched the literature using the search strings “antidepressants AND/in chronic pain,” “safety of antidepressants in chronic pain,” and “side effects of antidepressants in chronic pain” using the databases of the National Center for Biotechnology Information, the National Library of Medicine (MEDLINE), Google Scholar, and the Cochrane Central Register of Controlled Trials. Our literature search included studies from the first data available until the last search conducted in October 2015. However, due to the fact that treatment regimens changed over the last decades, studies published earlier than 1995 were excluded. Language restriction was applied including only articles in German and English.

The retrieved abstracts were stratified according to their relevance to the subject, and the full text of articles on the use of antidepressants in the treatment of chronic pain was retrieved. Additional articles were identified by cross-searching of the bibliographies of these publications. Case reports were excluded from the analysis. Trials that were included had to be conducted to study the use of antidepressants in the treatment of chronic pain and at least had to report on adverse effects of the treatment.

### Study Selection Criteria

According to the PICO guideline, we included an original study in our meta-analysis if the following eligibility criteria were met.

#### Population of Interest

Population of interest included patients with chronic pain being neuropathic, inflammatory/joint-related, or non-inflammatory/non-neuropathic pain. Pain conditions included in the analyses are detailed in Appendix S App-1 in Supplementary Material.

#### Intervention

The exposure variable (i.e., intervention) was defined as any antidepressant that was used for treatment of chronic pain. Specific antidepressants were only included in our meta-analysis when at least two RCT reporting adverse effects of the corresponding antidepressant were available. The study design was restricted to randomized placebo-controlled trials. Both constant and incremental dosing protocols were accepted. Both single and adjuvant use of antidepresants were accepted.

#### Control

The control group consisted of patients with chronic pain treated with placebo.

#### Outcome

Outcome parameters comprised any side effects that patients experienced during the follow-up period and tolerability that was defined as study discontinuation related to antidepressant therapy. We included only studies that reported quantitative data on adverse effects. We did not differentiate whether outcome data were reported as primary or secondary variable. In addition, for each included antidepressant, we analyzed pre-defined side effects and their relative risk of occurrence.

### Data Extraction

We extracted data on the number of included patients, the drug used as therapy and the occurrence side effects. The data collection and assessment of methodological quality were conducted as previously reported (8). The conduct and reporting were in accordance with the Quality of Reporting of Meta-Analyses statement.

### Statistical Analysis

The meta-analysis was performed using the statistical software R (©The R Foundation). Differences in incidences of overall adverse effects, withdrawal due to adverse effects, and specific adverse effects between the respective study arms were subsumed as pooled risk differences (RDs) or risk ratios (RRs) with 95%-confidence intervals (CIs) based on the random effects model of DerSimonian and Laird (9, 10). In addition, if sufficient metadata were available, 95% prediction intervals were reported. Prediction intervals estimate the range of effects expected to occur in future individual studies within the same study population. Complementary effect heterogeneity assessments were performed using forest plots and the inconsistency statistics (I^2^). Heterogeneity was evaluated by an analysis of the comparability of the following items: the number of patients, the grade or stage of disease, and the type of applied drug. In case of two drug doses per drug and trial, analysis was performed as two separate trials using the same placebo group. Conclusions regarding presence of evidence of effects were based on confidence and prediction interval limits rather than on statistical tests.

Inconclusive evidence occurred if the observed effect was close to RR = 1 and both CI limits would exceed clinically relevant margins of RR = 0.8 and RR = 1.2 (marked as: ±). Hence, intervals not exceeding either margin were interpreted as being supportive for evidence of possible RDs in the respective direction (marked as ++ or−−). Strongest evidence for an effect was provided if the associated prediction intervals met the same criterion (marked as +++ or −−−). In situations where RR estimates would be clinically relevant (exceeding the pre-specified margins), however, with wide CI limits exceeding both relevance margins, weak evidence for an effect was indicated (marked as + or −). Evidence of absence of an effect was indicated if the CI for the RR would exclude both margins (marked as 0).

## Results

### Literature Search

The MEDLINE search using above mentioned search terms revealed 1,975 articles published between 1982 and 2015. Of 1,613 articles in humans, 255 involved clinical trials whereas 158 were designed as RCT. No additional articles than those retrieved with the National Center for Biotechnology Information/National Library of Medicine databases were found using Google Scholar or Cochrane Central Register of Controlled Trials.

**Figure 1:**
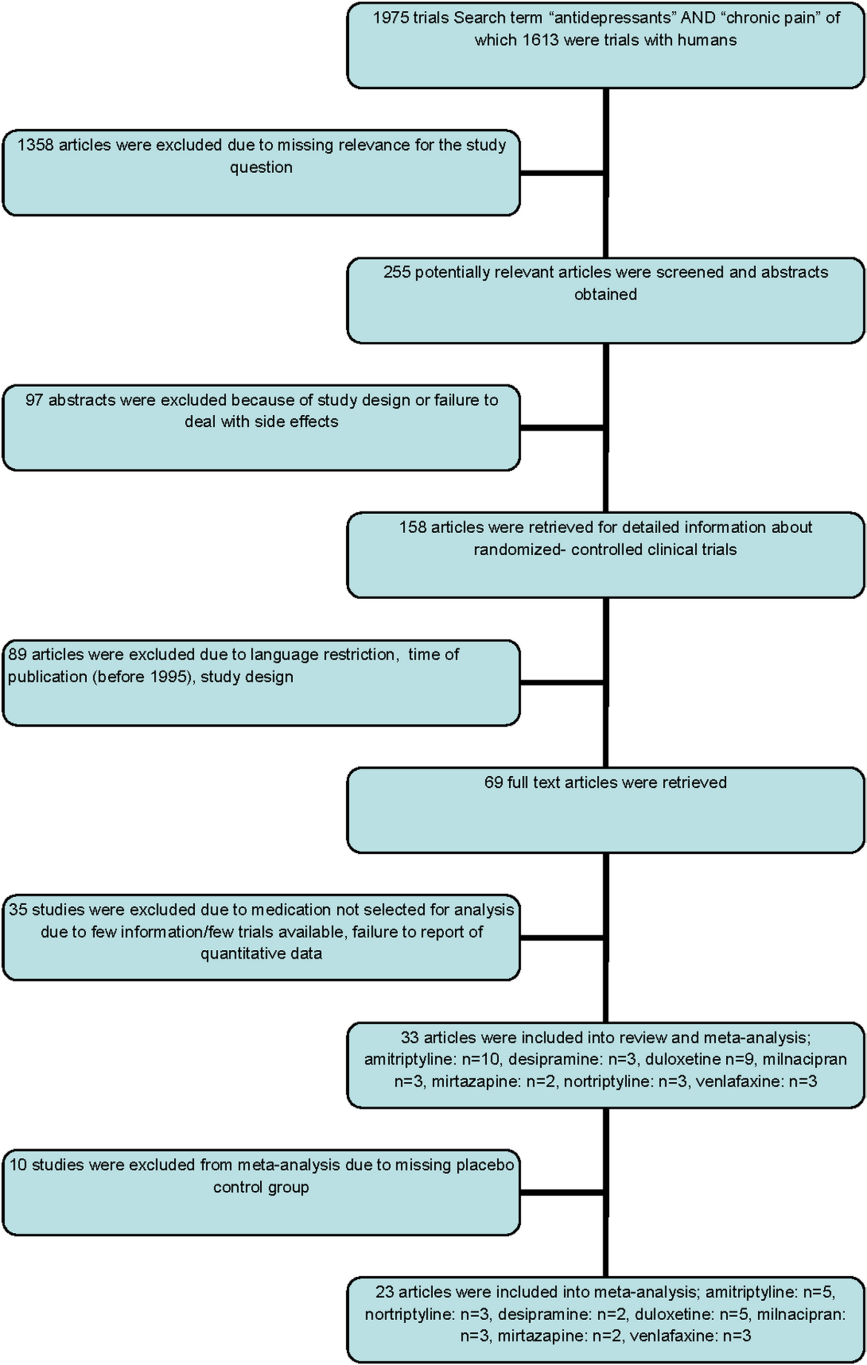
Flow chart of selection of trials included in the meta-analyses.

One study was excluded because of using the identical study population for two trials with different study aims. Another study was excluded because study population was restricted to children. Of 158 studies, 69 eventually met our selection criteria, and the full text articles of these were retrieved. Further selection excluded 36 studies without reporting quantitatively about occurrence of adverse effects. Finally, 33 clinical trials reporting on adverse effects were included in our quantitative data analysis. Ten trials had to be excluded for meta-analyses due to missing placebo control group, and 23 studies were included in the meta-analyses (Figure 1).

### Adverse Effects under Treatment with Antidepressants: Studies Included in the Systematic Review and Meta-analysis

Studies that met our inclusion criteria for systematic review and meta-analysis comprised clinical trials on amitryptiline, nortriptyline, desipramine, milnacipran, venlafaxine, duloxetine, mirtazapine, and fluoxetine. Tables S1 and S2 in Supplementary Material provide an overview of included antidepressants and their pharmacodynamic and pharmacokinetic characteristics. Table 1 provides an overview of included studies and their design.

**Table 1:** Placebo-controlled randomized clinical trials comparing antidepressants with placebo in the treatment of chronic pain.

Goldman et al. (11)		Amitriptyline (25 mg/day)	6 weeks	Arm pain due to repetitive use	1. *n* = 59	1. *n* = 52	1. *n* = 31/59 (52%)	1. *n* = 2/59 (3.4%)	Drowsiness
					3. *n* = 59	3. *n* = 55	3. *n* = *16*/59 (27%)	3. *n* = 0	

Cardenas et al. (12)	RCT	Amitriptyline (10–125 mg/day)	6 weeks	Pain of spinal cord injury	1. *n* = 44	1. *n* = 37	1. *n* = 43/44 (97%)	1. *n* = 7/44 (16%)	Dry mouth, drowsiness, urinary difficulty, constipation, sweating, headache, irritability, palpitations, diarrhea, blurred vision
	DB				3. *n* = 40	3. *n* = 38	3. *n* = 36/40 (90%)	3. *n* = 2/40 (5%)	

Rani et al. (13)	RCT	1. Amitriptyline (25 mg/day)	4 weeks	Chronic pain syndrome (27 low-back pain, 16 osteoarthritis, 8 FM, 8 rheumatoid arthritis)	1. *n* = 20		1. *n* = 31		Dizziness, constipation, headache, palpitations, edema, gastritis, thirst, tachycardia
	DB	2. Fluoxetine (20 mg/day)			2. *n* = 21		2. *n* = 22		
					3. *n* = 18		3. *n* = 12		

Bendtsen et al. (14)	RCT	Amitriptyline (25–75 mg/day) vs. citalopram (20 mg/day)	32 weeks (8 weeks tx, 2 weeks wash-out)	Tension-type headache	1. *n* = 40		1. *n* = 33/40 (82%)	1. *n* = 1/40 (2%)	Dry mouth, drowsiness, dizziness, weight gain, nausea, constipation
	DB				2. *n* = 40		2. *n* = 15/40 (37%)	2. *n* = 0	
	3w-CO				3. *n* = 40		3. *n* = 15/40 (37%)	3. *n* = 0	

Boline et al. (15)	RCT	Amitriptyline (10–30 mg/day) vs. spinal manipulation	6 weeks	Tension-type headache	1. *n* = 75	1. *n* = 56	1. *n* = 46/75 (61%)	1. *n* = 5/75 (7%)	Drowsiness, dry mouth, weight gain
					3. *n* = 75	3. *n* = 70	3. *n* = 3/75 (4%)	3. *n* = 0	

Khoromi et al. (16)	RCT	Nortriptyline (25–100 mg/day) vs. morphine	9 weeks (5 weeks dose escalation, 2 weeks maintain-ance, 2 weeks tapering)	Chronic lumar root pain	1. *n* = 13	1. *n* = 7	1. *n* = 9/13 (37%)	1. *n* = 2/13 (15%)	Constipation, dry mouth, drowsiness, dizziness, somnolence, headache, insomnia, weight gain, urinating difficulty, abdominal pain, heart burn
	DB				2. *n* = 15	2. *n* = 9	2. *n* = 14/15 (93%)	2. *n* = 5/15 (7%)	
	CO				3. *n* = 13	3. *n* = 5	3. *n* = 4/5 (31%)	3. *n* = 4/13 (31%)	
	SC								

Holroyd et al. (17)	RCT	Nortriptyline (75 mg/day or amitriptyline 100 mg/day)	8 weeks	Chronic tension-type headache	1. *n* = 97	1. *n* = 53	1. *n* = 78/97 (80%)	1. *n* = 1/97 (1%)	Drowsiness, dry mouth, dizziness, constipation, weight gain, abdominal pain, increased appetite
	DB				3. *n* = 90	3. *n* = 48	3. *n* = 27/90 (23%)	3. *n* = 3/90 (3%)	

Atkinson et al. (18)	RCT	Nortriptyline (escalating 25–50–100 mg/dl)	8 weeks	Chronic low-back pain	1. *n* = 38	1. *n* = 28	1. *n* = 28/38 (74%)	1. *n* = 4/38 (10%)	Dry mouth, insomnia, sedation, orthostatic hypotension, constipation, sweating, palpitations
	DB				3. *n* = 40	3. *n* = 29	3. *n* = 28/40 (70%)	3. *n* = 0/40	

Atkinson et al. (19)	RCT	Desipramine (50–110–150 ng/ml levels) vs. Fluoxetine	12 weeks	Chronic back pain	1. *n* = 52	1. *n* = 30	1. *n* = 19/30 (63%)	1. *n* = 17/52 (33%)	Decreased salivation, constipation, loss of libido
	DB				2. *n* = 43	2. *n* = 31	2. *n* = 16/31 (53%)	2. *n* = 3/43 (7%)	
	SC				3. *n* = 26	3. *n* = 22	3. *n* = 3/22 (14%)	3. *n* = 1/26 (4%)	

Wallace et al. (20)	RCT	Desipramine (escalating 50–300 mg/day)	35 weeks (14 weeks tx)	Capsicain-induced allodynia	1. *n* = 12		1. *n* = 5/12 (42%)	1. *n* = 1/13 (8%)	Constipation, dry mouth, vomiting, drowsiness, dizziness, sweating, headache, insomnia, shaking
	DB				3. *n* = 12		3. *n* = 0/12	3. *n* = 0/13	
	CO								

Clauw et al. (21)	RCT	Milnacipran (100 mg/day)	12 weeks	Fibromyalgia	1. *n* = 100	1. *n* = 75	1. *n* = 47/100 (47%)	1. *n* = 2/100 (2%)	Nausea, vomiting, headache, sinusitis/nasopharyngitis, respiratory infection, fatigue, fall, arthralgia, edema
	DB				3. *n* = 50	3. *n* = 31	3. *n* = 29/50 (58%)	3. *n* = 0	
	MC								

Mease et al. (22)	RCT	Milnacipran (100 mg/day; 200 mg/day)	27 weeks	Fibromyalgia	1. *n* = 224	1. *n* = 128	1. *n* = 188/224 (84%)	1. *n* = 44/224 (20%)	Nausea, vomiting, dizziness, constipation, headache, tachycardia, palpitations, hot flush, dry mouth, sweating, insomnia, sinusitis, nasopharyngitis, respiratory infection, diarrhea
	DB				2. *n* = 441	2. *n* = 239	2. *n* = 400/441 (90%)	2. *n* = 119/441 (27%)	
	MC				3. *n* = 223	3. *n* = 145	3. *n* = 190/223 (85%)	3. *n* = 23/223 (10%)	

Clauw et al. (23)	RCT	Milnacipran (100 mg/day; 200 mg/day)	15 weeks	Fibromyalgia	1. *n* = 401	1. *n* = 399	1. *n* = 358/401 (90%)	1. *n* = 78/401 (20%)	Nausea, vomiting, dizziness, constipation, headache, hypertension, tachycardia, palpitations, hot flush
	DB				2. *n* = 401	2. *n* = 396	2. *n* = 346/401 (87%)	2. *n* = 94/401 (24%)	
	MC				3. *n* = 405	3. *n* = 401	3. *n* = 317/405 (79%)	3. *n* = 38/405 (9%)	

Ozyalcin et al. (24)	RCT	Venlafaxine XR (75 mg/day; 150 mg/day)	8 weeks	Migraine	1. *n* = 20	1. *n* = 15	1. *n* = 20/20 (100%)	1. *n* = 3/20 (15%)	Nausea, vomiting, drowsiness, dizziness, somnolence, mydriasis, jaw spasm, constipation, loss of libido, dry mouth, sweating, loss of appetite, sedation
					2. *n* = 21	2. *n* = 17	2. *n* = 20/21 (95%)	2. *n* = 3/21 (14%)	
					3. *n* = 19	3. *n* = 17	3. *n* = 10/19 (53%)	3. *n* = 0/19	

Yucel et al. (25)	RCT	Venlafaxine XR (75 mg/day; 150 mg/day)	8 weeks	Neuropathic pain	1. *n* = 20	1. *n* = 19	1. *n* = 9/20 (45%)	1. *n* = 1/20 (5%)	Nausea, vomiting, dizziness, somnolence
	DB				2. *n* = 20	2. *n* = 17	2. *n* = 14/20 (70%)	2. *n* = 3/20 (15%)	
					3. *n* = 20	3. *n* = 19	3. *n* = 11/20 (55%)	3. *n* = 1/20 (5%)	

Forssell et al. (26)	R	Venlafaxine (37.5 mg 1–2×/day)	10 weeks (4 weeks tx)	Atypical facial pain	1. *n* = 30	1. *n* = 20	1. *n* = 18/30 (60%)	1. *n* = 6/30 (20%)	Nausea, constipation, dry mouth, sweating, loss of appetite, urinating difficulty, fatigue, nightmares, headache, palpitations
	DB				3. *n* = 30	3. *n* = 20	3. *n* = 18/30 (60%)	3. *n* = 2/30 (7%)	
	CO								

Chappell et al. (27)	RCT	Duloxetine (60–120 mg/day)	13 weeks	Osteoarthritis of the knee	1. *n* = 128	1. *n* = 93	1. *n* = 64 (50%)	1. *n* = 24/128 (19%)	Nausea, constipation, hyperhidrosis
	DB				3. *n* = 128	3. *n* = 111	3. *n* = 41 (32%)	3. *n* = 7/128 (5%)	

Ho et al. (28)	RCT	Duloxetine 60 mg	2 h before surgery; d1 after surgery	Knee replacement surgery	1. *n* = 23	*N* = 0	1. *n* = 6/23 (26%)	*N* = 0	Nausea, dizziness, headache, pruritus
	DB				3. *n* = 24		3. *n* = 12/24 (50%)		

Skljarevski et al. (30)	RTC	Duloxetine (60 mg/day)	12 weeks	Chronic low-back pain	1. *n* = 198	1. *n* = 147	1. *n* = 125/198 (63%)	1. *n* = 30/198 (15%)	Nausea, dizziness, somnolence, headache, constipation, dry mouth, vertigo, myocardial infarction, muscular weakness, asthma
	DB				3. *n* = 203	3. *n* = 156	3. *n* = 112/203 (55%)	3. *n* = 11/203 (5%)	

Skljarevski et al. (31)	RCT	Duloxetine (60–120 mg/day)	13 weeks	Chronic low-back pain	1. *n* = 115	1. *n* = 84	1. *n* = 65/115 (57%)	1. *n* = 16/115 (14%)	Nausea, dizziness, somnolence, headache, dry mouth, fatigue, diarrhea, Hyperhidrosis, TIA, osteoarthritis, constipation
	DB				3. *n* = 121	3. *n* = 98	3. *n* = 58/121 (48%)	3. *n* = 7/121 (6%)	

Skljarevski et al. (29)	RCT	Duloxetine [(1.) 20mg/day, (2.) 60 mg/day or (2*.) 120 mg/day]	13 weeks	Chronic low-back pain	1. *n* = 59	1. *n* = 43	1. 64.4%	1. *n* = 9/59 (15%)	TIA, myocardial infarction, dyspnoe, weakness, diarrhea, dizziness
	DB				2. *n* = 116	2. *n* = 80	2. 67.2%	2. *n* = 17/116 (15%)	
					2*. *n* = 112	2*. *n* = 62	2*. 72.3%	2*. *n* = 27/112 (24%)	
					3. *n* = 117	3. *n* = 82	3. 59%	3. *n* = 10/117 (9%)	

Bendtsen and Jensen (33)	RCT	Mirtazapine (15–30 mg/day)	18 weeks (8 weeks tx)	Chronic tension-type headache	1. *n* = 24		1. *n* = 24/24 (100%)	1. *n* = 2/24 (8%)	Drowsiness, dizziness, weight gain, dry mouth, increased appetite, edema, sleep disturbances, nausea, concentrations difficulties, irritability
	DB				3. *n* = 24		3. *n* = 18/24 (75%)	2. *n* = 0/24	
	CO								

Arnold et al. (32)	RCT	Mirtazapine (30 mg/day)	1 single dose	Healthy patients	1. *n* = 10		1. *n* = 9/10 (90%)		Sedation, dizziness, dry mouth, unpleasant metallic taste, global weakness, swallowing difficulty
	DB				3. *n* = 10		3. *n* = 2/10 (20%)		
	CO								

Atkinson et al. (19)	RCT	Fluoxetine (100, 200, 400 ng/ml) vs. desipramine	12 weeks	Chronic back pain	1. *n* = 52	1. *n* = 30	1. *n* = 16/31 (53%)	1. *n* = 3/43 (7%)	Loss of libido, dry mouth
	DB				2. *n* = 43	2. *n* = 31	2. *n* = 19/30 (63%)	2. *n* = 17/52 (33%)	
	SC				3. *n* = 26	3. *n* = 22	3. *n* = 3/22 (14%)	3. *n* = 1/26 (4%)	

Rani et al. (13)	RCT	Fluoxetine (20 mg/day)	4 weeks	Chronic pain syndrome (27 low-back pain, 16 osteoarthritis, 8 FM, 8 rheumatoid arthritis)	1. *n* = 21		1. *n* = 22	*N* = 0	Nausea, dizziness, headache, palpitations, edema, gastritis, loss of appetite, breathlessness
	DB				2. *n* = 20		2. *n* = 31		
					3. *n* = 18		3. *n* = 12		

*n, number; RCT, randomized-controlled trial; DB, double-blind; MC, multicenter; SC, single center; CO, crossover; 3w-CO, 3-way crossover; tx, treatment*.

#### Amitriptyline

Goldman et al. compared amitriptyline (25 mg/day) and placebo in the treatment of chronic arm pain in 118 patients. 107 patients completed the trail. Overall adverse effects occurred in 31/59 patients (52%) under amitriptyline and in 16/59 patients (27%) under placebo [RD +25% CI: +6%; +42%]. Withdrawal due to adverse effects was necessary in the amitriptyline-group in 2/59 patients (3%) [RD +3% CI: −4%; +12%]. The main side effect was drowsiness (11). Similarly, Cardenas et al. reported adverse effects of amitriptyline (10–125 mg/day) and placebo in the treatment of pain related to spinal cord injury in 84 patients. Overall adverse effects occurred in 43/44 patients (97%) in the amitriptyline group and 36/40 patients (90%) in the placebo group [RD +7% CI: −5%; +22%]. Withdrawal due to adverse effects was necessary in 7/44 patients (16%) in the amitriptyline and 2/40 patients (5%) in the placebo group [RD +11% CI: −5%; +26%]. Drowsiness, constipation, and dry mouth were the most frequent adverse effects. Less often, headache, palpitations, and irritability as well as blurred vision were reported (12). Another placebo-controlled trial, published by Rani et al., compared adverse effects of amitriptyline (25 mg/day), fluoxetine (20 mg/day), and placebo in 59 patients with chronic pain syndromes. Reported adverse effects occurred 31 times in 20 patients under amitriptyline, 22 times in 21 patients under fluoxetine, and 12 times in 18 patients under placebo. Withdrawal due to adverse effects was not necessary. Reported adverse effects of amitriptyline were mainly dizziness, constipation, headache, and palpitations (13). Similarly, Bendtsen et al. compared the treatment of amitriptyline (75 mg/day), citalopram (20 mg/day), and placebo in 40 patients under crossover conditions. Under treatment with amitriptyline, 33/40 patients (82%) reported adverse effects, whereas 15/40 patients (37%) under treatment with citalopram and 15/40 patients (37%) under placebo [RD +45% CI: +22%; +62%] reported adverse effects, 1/40 (2%) patient had to be withdrawn due to adverse effects under amitriptyline [RD +2% CI: −9%; +15%]. Drowsiness and dry mouth were the main adverse effects, followed by dizziness and constipation and gain of body weight (14). Boline et al. compared amitriptyline with spinal cord manipulation in the treatment of chronic tension-type headaches in 150 patients. 46/75 patients (61%) vs. 3/75 patíents (4%) reported about adverse effects in the amitriptyline group [RD +57% CI: +43%; +68%]. Withdrawal due to adverse effects was necessary in 5/75 (7%), while no withdrawal was necessary in the spinal manipulation group [RD +7% CI: −1%; +15%]. Main adverse effects of amitriptyline were drowsiness and dry mouth (15).

#### Nortriptyline

Khoromi et al. compared nortriptyline (25–100 mg/day), morphine (15–90 mg/day), and active placebo benztropine in the treatment of lumbar radicular pain in patients. Overall adverse effects were reported in 9/13 patients (68%) in the nortriptyline group, in 14/15 patients (93%) in the morphine group, and 6/13 patients (50%) in the placebo group [RD +23% CI: −18%; +56%]. Withdrawal due to adverse effects was necessary in 2/13 patients (15%) in the nortriptyline group, 5/15 patients (7%) in the morphine group, and 1/14 patients (7%) in the placebo group [RD +8% CI: −23%; +40%]. Main adverse effects in the nortriptyline group were constipation and dry mouth, followed by somnolence, dizziness, drowsiness, headache, insomnia, weight gain, and heart burn (16). Comparison of amitryptiline (100 mg/day) or nortriptyline (75 mg/day) and placebo in the treatment of chronic tension-type headaches was performed by Holroyd et al. Noteworthy, authors analyzed effects and adverse effects of amitriptyline and nortriptyline as one “treatment” group compared to the placebo group. 78/97 patients (80%) in the treatment group had adverse effects as opposed to 27/90 patients (23%) in the placebo group [RD +50% CI: +36%; +62%]. Withdrawal due to adverse effects was necessary in 1/97 (1%), but 3/90 (3%) in the placebo group [RD +2% CI: −3%; +9%]. The main adverse effects were drowsiness (44%) and dry mouth (53%) compared to placebo group with 11 and 13%, respectively (17). Atkinson et al. compared nortriptyline at escalating doses (25 up to 100 mg/day) with inert placebo in the treatment of chronic back pain in 78 patients. 57 patients completed the study. In the treatment group 28/28 patients (100%) analyzed patients participating in the trail reported adverse effects whereas 28/29 patients (97%) reported adverse effects under placebo [RD +4% CI: −18%; +24%]. However, withdrawal due to adverse effects was necessary in 4/38 patients (10%) under therapy with nortriptyline, but was not necessary in the placebo group [RD +10% CI: −2%; +26%]. Main adverse effects were constipation, dry mouth and insomnia, sedation, sweating, palpitations, and orthostatic hypotension in the nortriptyline group. Adverse effects were reported more frequently in the nortriptyline group (18).

#### Desipramine

Atkinson et al. compared the treatment of desipramine, fluoxetine, and placebo for the treatment of chronic low-back pain in 121 patients. Doses were adjusted to measured blood concentration of the specific drugs. Three different subgroups according to blood concentrations of desipramine (levels of 50, 110, and 150 ng/ml) and fluoxetine (blood levels of 100, 200, and 400 ng/ml) were included. Overall adverse effects were reported in 19/39 patients (63%) for desipramine, 16/31 patients (53%) for fluoxetine, and 3/22 patients (14%) for placebo [RD +50% CI: +20%; +69%]. The study was completed by 83 out of 121 patients. Withdrawal due to adverse effects was necessary in 17/52 patients (33%) under desipramine and in 1/26 patients (4%) under placebo [RD +29% CI: +7%; +44%]. Adverse effects were similar for patients with desipramine blood concentrations lesser or greater than 60 ng/ml. The main adverse effects for desipramine were dry mouth and constipation, whereas fluoxetine mainly led to sexual disturbances (19). In a crossover trial by Wallace et al., allodynia was induced by capsicain in 12 healthy patients and treatment with desipramine at escalating doses (50 up to 300 mg/day within 14 days) was compared with placebo. Adverse effects occurred in 5/12 (42%) patients vs. 0/12 in the placebo group [RD +42% CI: +2%; +71%]. One withdrawal due to adverse effects [1/13; (7.6%)] was reported in the desipramine group [RD +8% CI: −21%; +38%]. The two most common adverse effects were dry mouth and drowsiness. Fewer adverse effects were sweating, headache, shaking, insomnia, nausea, vomiting, and constipation (20).

#### Milnacipran

Clauw et al. analyzed the treatment of milnacipran at 100 mg/day in the treatment of fibromyalgia in 150 patients. Overall adverse effects were reported in 47/100 (47%) patients in the milnacipran group and in 29/50 patients (58%) in the placebo group [RD +11% CI: −7%; +28%]. Main adverse effects were nausea, vomiting, headache, nasopharyngitis and respiratory infections, fatigue, fall, arthralgia, and edema. Similar adverse effects were also reported in the placebo group. Adverse effect-related discontinuation was necessary in 2/100 patients (2%) under milnacipran vs. 0 in the placebo group [RD +2% CI: −7%; +8%] (21). Similarly, Mease et al. compared milnacipran at 100 and 200 mg/day with placebo in the treatment of fibromyalgia in 888 patients. The study was completed by 512 patients. Overall, adverse effects were reported in 188/224 patients (84%) in the milnacipran 100 mg-group, 400/441 (90%) in the 200 mg-milnacipran group, and 190/223 patients (85%) in the placebo group [RD +1% CI: −6%; +8%] and [RD +6% CI: 0%; +12%]. Withdrawal due to adverse effects was necessary in 44/224 patients (20%) in the low-dose milnacipran group, in 119/441 patients (27%) in the high dose milnacipran group, and in 23/223 patients (10%) in the placebo group [RD +10% CI: 3%; +17%] and [RD +17% CI: +11%; +23%], respectively. Slow titration of dose and duration to mitigate these events was done in all cases. Main adverse effects were nausea, vomiting, dizziness and constipation, headache, tachycardia, palpitations, hot flush, dry mouth, and sweating (22). A multicentric 3-arm study by Clauw et al. compared milnacipran at 100 and 200 mg/day with placebo in the treatment of 1,207 patients with fibromyalgia. Adverse effects were reported in 358/399 patients (90%) in the 100 mg-milnaciprane group, 346/396 patients (87%) in the 200 mg-milnacipran group, and in 316/401 patients (79%) in the placebo group [RD +11% CI: +6%; +16%] and [RD +6% CI: +3%; +14%]. Withdrawal due to adverse effects was reported in 78/399 patients (20%), 94/396 patients (24%), and 38/401 patients (9%) patients for the 100 mg-, 200 mg-, and placebo group, respectively [RD +10% CI: +5%; +15%] and [RD +14% CI: +9%; +20%], respectively (23).

#### Venlafaxine

Ozyalcin et al. compared venlafaxine at doses of 75 and 150 mg/day with placebo in the treatment of migraine in 60 patients. 49 completed the trial. Overall adverse effects were reported in 20/20 patients (100%) for the venlafaxine 75 mg/day, in 20/21 patients (95%) for the venlafaxine 150 mg/day, and 10/19 patients (53%) for the placebo group [RD +47% CI: +17%; +70%] and [RD +43% CI: +12%; +66%], respectively. Withdrawal due to adverse effects was necessary in 3/20 patients (15%) for the venlafaxine 75 mg/day and 3/21 patients (14%) in the venlafaxine 150 mg/day group, but not in the placebo group [RD +15% CI: −9%; +39%] and [RD +14% CI: −9%; +37%]. The main adverse effects were nausea, vomiting, drowsiness, and insomnia (24). Yucel et al. compared venlafaxine at doses of 75 and 150 mg/day with placebo in the treatment of neuropathic pain in 60 patients. 55 patients completed the trial. Overall adverse effects were reported in 9/20 patients (45%) for the 75 mg, in 14/20 patients (70%) for the 150 mg, and 11/20 patients (55%) for the placebo group [RD +10% CI: −2%; +40%] and [RD +15% CI: −7%; +44%]. Withdrawal due to adverse effects was necessary in 1/20 patients (5%), 3/20 patients (15%), and 1/20 patients (5%) for the venlafaxine 75 mg/day, the venlafaxine 150 mg/day, and the placebo group, respectively [RD 0% CI: −2%; +22%] and [RD +10% CI: −15%; +34%]. Adverse effects were nausea, vomiting, dizziness, and somnolence (25). Forssell et al. compared venlafaxine at 37.5 mg 1–2×/day and placebo for the treatment of atypical facial pain in 30 patients in a double-blind crossover trial. Adverse effects occurred in 18/30 patients (60%) in the venlafaxine group and in 18/30 patients (60%) in the placebo group. Withdrawal due to adverse effects was necessary in 6/30 patients (20%) during treatment with venlafaxine and 2/30 patients (7%) under placebo [RD +13% CCI: −7%; +33%]. There was no difference regarding adverse effects between both groups (26).

#### Duloxetine

Chappell et al. compared 60–120 mg/day duloxetine with placebo in the treatment of chronic pain due to osteoarthritis in 256 patients. Only 111(87%) patients in the placebo and 93 (73%) in the duloxetine group completed the study. Patients treated with duloxetine had greater improvement in pain scores, but also showed more adverse effects than placebo-treated patients [64/128(50%) vs. 41/128(32%)] [RD +18% CI: +5%; +30%]. Withdrawal due to adverse effects was necessary in 24/128 patients (19%) under duloxetine vs. 7/128 patients (5%) under placebo [RD +13%: +5%; +22%](27). Ho et al. analyzed the potential of duloxetine to reduce morphine requirements after knee-replacement surgery in 50 patients. Patients received either duloxetine 60 mg/day or placebo. 47 patients were included in final analysis. Most common adverse effects in the duloxetine group were nausea and vomiting, followed by dizziness and headache. Adverse effects were more common in the placebo group 12/24 (50%) than in the duloxetine group 6/23 (26%) [RD +24% CI: −7%; +49%]. However, this comparison included all noted adverse effects irrespectively of their severity or mechanism. One patient of the duloxetine group died 6 months after surgery due to pneumonia. This death was not drug-related. Withdrawal due to adverse effects was not necessary in either group (28). In a study by Skljarevski et al., duloxetine at doses of 20, 60, or 120 mg/day was compared to placebo in the treatment of chronic low-back pain in 404 patients of which 267 completed the trial. Discontinuation due to adverse effects was seen more frequently in the 120 mg duloxetine group (24.1% of the patients) compared to placebo (8.5%) (29). Similarly, another study by the same group compared duloxetine dosages ranging from 60 to 120 mg/day with placebo in the treatment of chronic lower back pain. About twice as many patients discontinued because of adverse events in the duloxetine group (13.9%) than in the placebo group (5.8%). The most common reported adverse effects were nausea, dry mouth, fatigue, diarrhea, sweating, dizziness, and constipation (30). In their third randomized placebo-controlled trial, Skljarevski et al. analyzed 401 patients treated for CLBP with 60 mg/day duloxetine or placebo. More patients discontinued due to adverse effects in the duloxetine group (15.2%) compared to the placebo group (5.4%). Overall adverse effects were more frequent in the duloxetine group. Most frequent adverse effects were nausea and dry mouth (31).

#### Mirtazapine

In in a double-blind crossover study in healthy subjects 30 mg/day mirtazapine or placebo was administered. Adverse effects occurred in 9/10 patients (90%) under mirtazapine and in 2/10 patients (20%) under placebo [RD +70% CI: +19%; +89%]. Reported adverse effects were dizziness, dry mouth, transient sedation, global weakness, unpleasant metallic taste, and swallowing difficulty under treatment with mirtazapine (32). Bendtsen and Jensen analyzed mirtazapine in doses of 15–30 mg/day in a double-blind, placebo-controlled crossover study in 24 patients with tension-type headache. Overall, adverse effects were reported in 24 of 48 patients (50%) receiving mirtazapine compared to 18 of 48 patients (38%) treated with placebo [RD +13% CI: −8%; +32%]. Withdrawal due to adverse effects was necessary in two patients during treatment with mirtazapine (4%), but none within the placebo group [RD +4% CI: −6%; +15%]. Main adverse effects were drowsiness, dizziness, weight gain, and dry mouth (33).

#### Fluoxetine

Atkinson et al. compared desipramine, fluoxetine, and placebo for the treatment of chronic low-back pain in 121 patients. Overall adverse effects were reported in 16/31 patients (53%) for fluoxetine and 3/22 patients (14%) in the placebo group [RD +38% CI: +9%; +58%]. Withdrawal due to adverse effects was necessary in 3/43 patients (7%) under fluoxetine vs. 1/26 patients (4%) under placebo [RD +3% CI: −15%; +17%]. Adverse effects of fluoxetine were mainly sexual disturbances (19). A study by Rani et al. compared adverse effects of amitriptyline (25 mg/day), fluoxetine (20 mg/day), and placebo in 59 patients with chronic pain syndromes. Reported adverse effects occurred in 21 patients under fluoxetine and in 18 patients under placebo. Withdrawal due to adverse effects was not necessary in both groups. Reported adverse effects of fluoxetine were mainly nausea, dizziness, headache, palpitations, edema, gastritis, loss of appetite, and breathlessness (13).

### Adverse Effects under Treatment with Antidepressants: Studies Included Only in the Systematic Review

Studies that matched our inclusion criteria for review, but were not included in the meta-analyses due to absence of a placebo group comprised trials on amitryptiline, desipramine, and duloxetine.

#### Amitriptyline

Liu et al. compared in the therapy of neuropathic pain amitriptyline with nortriptyline and an untreated control group. The study population included 228 patients. While no adverse effects and no withdrawals were reported in the control group, overall adverse effects occurred in 25/89 patients (28%) under amitriptyline and in 31/106 patients (29%) under nortriptyline. Withdrawal due to adverse effects was reported in 16/89 patients (18%) in the amitriptyline group vs. 13/106 patients (12%) under nortriptyline. The main side effect in both groups was sedation, followed by dizziness, drowsiness and dry mouth, and gain of body weight. There was no significant difference between both treatment groups in regard to adverse effects (34). Kalita et al. compared amitriptyline at escalating doses (12.5–50 mg/day) with pregabaline at escalating doses (75 mg 2×/day up to 300 mg 2×/day) in the treatment of low-back pain in 200 patients. Similar rates of overall adverse effects for amitriptyline (18/103 patients, 17%) and pregabaline (21/97 patients, 22%) were reported. Rates of withdrawal due to adverse effects were also similar (amitriptyline 11/103 patients, 11% and pregabaline 12/97 patients, 12%). Dry mouth and sedation were most frequently reported in the amitriptyline group (35). Magalhaes et al. compared the outcome of 72 patients with migraine treated with botulinum toxine type A (BTX) injections or systemic therapy with oral amitriptyline (25–50 mg/day). BTX injections were given once at defined points in the area of head and neck. Patients treated with amitriptyline showed higher incidences of constipation in 14/37 patients (38%) vs. 0/35 patients (0%), dry mouth in 16/37 patients (43%) vs. 5/35 patients (14%), body weight gain in 16/37 patients (43%) vs. 4/35 patients (11%), and somnolence in 19/37 patients (51%) vs. 1/35 patients (3%) patients (36). Jose et al. compared amitriptyline (10–50 mg/day) with lamotrigine (50–200 mg/day) in 46 patients with diabetic neuropathy in a crossover study. In this study, 33/46 patients (72%) developed adverse effects under treatment with amitriptyline as opposed to 11/46 patients (24%) under treatment with lamotrigine. Furthermore, 19/46 patients (41%) had to be withdrawn due to adverse effects in the amitriptyline group vs. 8/46 patients (17%) patients in the lamotrigine group. Dizziness, constipation and dry mouth, somnolence, and increased sleep was reported for amitriptyline and hot flush as well as increased creatinine levels for lamotrigien as major adverse effects (37). Rintala et al. compared the 8-week treatment with amitriptyline (50 mg, 3×/day), gabapentine (1,200 mg, 3×/day), and diphenhydramine (25 mg, 3×/day) in patients with neuropathic pain induced by spinal cord injuries in a crossover study design with 210 patients. Adverse effects were reported in 67/210 patients (32%) under treatment with amitriptyline, in 57/201 patients (28%) under treatment with gabapentine, and 43/205 patients (21%) patients under treatment with diphenhydramine as active control group. Main adverse effects of amitriptyline were drowsiness, constipation, dry mouth, dizziness, nausea, palpitations, and weight gain (38).

#### Desipramine

Walker et al. compared desipramine at 75 mg/day and fluoxetine at 20 mg/day in the treatment of chronic tension-type headache in 37 patients. The study was completed by 25 out of 37 patients. Adverse effects occurred in 6/19 patients (32%) of the desipramine group and 6/18 patients (33%) in the fluoxetine group. Withdrawal due to adverse effects was necessary in 4/19 patients (21%) under desipramine vs. 6/18 patients (33%) under fluoxetine. Reasons for withdrawal were drowsiness, fatigue, excessive sleepiness, malaise, nausea, weakness, faintness, insomnia, and headache. Patients taking fluoxetine discontinued the study medication for nausea, vomiting, and malaise (39).

#### Duloxetine

Leombruni et al. reported data of a comparative trial with 60 mg/day duloxetine and 1,500 mg/day l-carnitine in 65 female patients with FM of which 51/65 patients (78.5%) completed the trial. Both treatments led to improvement in pain and depression, but there were significant more adverse effects in the duloxetine group [8/29 (27%) patients vs. 0/22 patients] compared with the l-carnitine group. Reported adverse effects under duloxetine were nausea, anxiety, insomnia, and diarrhea. Out of 14 dropouts, 8 were due to duloxetine-related adverse effects (40). Giannantoni et al. compared additional therapy with 60 mg/day duloxetine + standard treatment with 0.4 mg/day tamsulosin + 320 mg/day palmetto and standard multidrug regimen with 0.4 mg/day tamsulosin + 320 mg/day palmetto in 38 patients with chronic pelvic pain syndrome. Withdrawal due to adverse effects was more frequent (4/19 patients, 20%) in the duloxetine group compared to the standard treatment with (0/19 patients, 0%) (41). Mazza et al. compared 60 mg/day duloxetine with 20 mg/day escitalopram in the treatment of CLBP in 85 patients of which 80 completed the study. Adverse effects were similar in both groups (15/41 patients, 36% under duloxetine vs. 14/39 patients, 36%) under escitalopram. Adverse effect-related treatment discontinuation was not necessary in both groups (42).

### Meta-analysis

Overall, 60 different adverse effects under therapy with antidepressants were reported in the studies included in the review and/or the meta-analyses. Reported side effects are listed in Table S2 in Supplementary Material. Meta-analyses revealed higher risks for overall adverse effects and side effect-related withdrawals under treatment with antidepressants compared to placebo. Specific adverse effects occurring under treatment with antidepressants of either subgroup were identified.

#### RRs and RDs for Overall Adverse Effects

**Figure 2:**
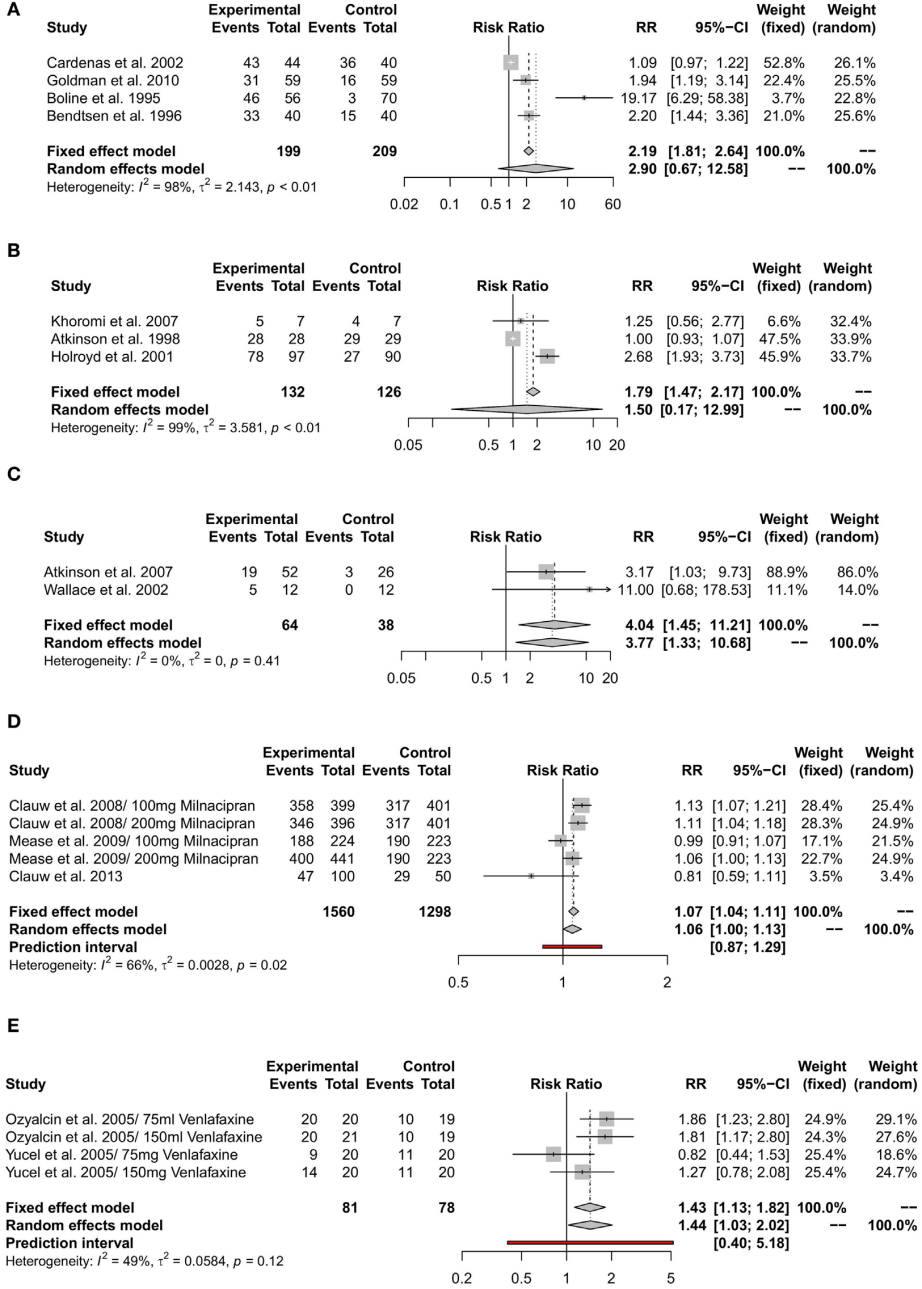

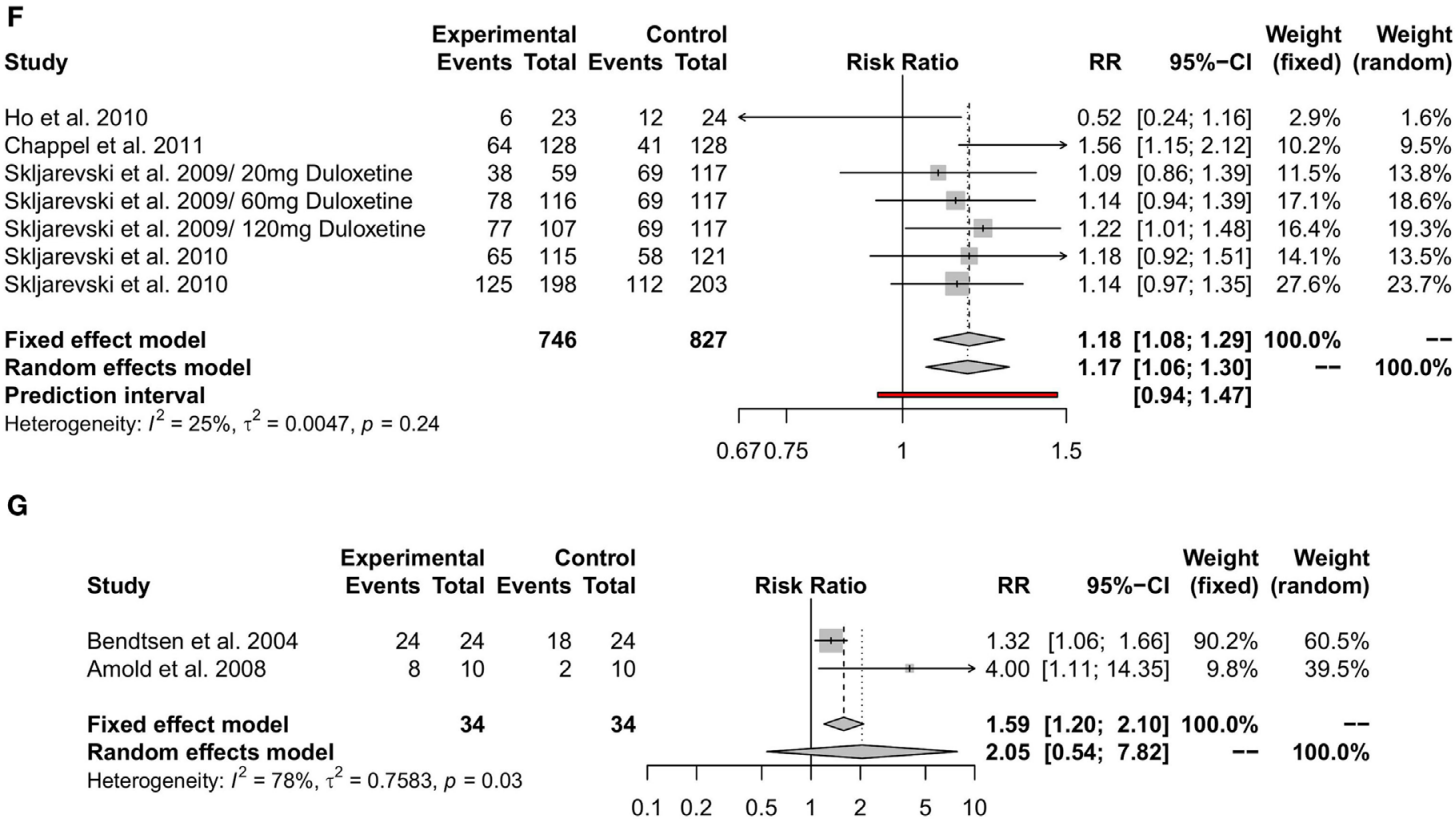
Meta-analyses of overall adverse effects under treatment with different antidepressants. RR = risk ratio; 95%-CI = 95% confidence interval; PI = prediction interval. Overall adverse effects under treatment with amitriptyline **(A)**, with nortriptyline **(B)**, with desipramine **(C)**, with milnacipran **(D)**, with venlafaxine **(E)**, with duloxetine **(F)**, and with mirtazapine **(G)**.

**Figure 3:**
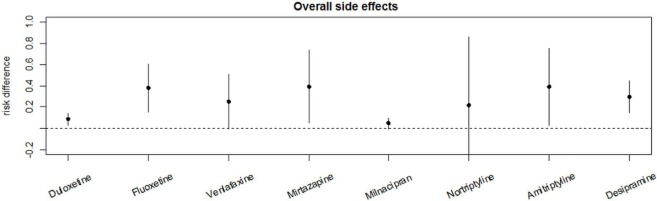
Comparative analysis for the placebo effect-adjusted risk of the overall adverse effects of the different analyzed antidepressants. Risk differences were used for placebo effect-adjusted risk.

Among TCAs, the RRs for overall adverse effects were 2.9 (95%-CI [0.67; 12.58]) under treatment with amitriptyline, 1.50 (95%-CI [0.17; 12.99]) under nortriptyline, and 3.77(95%-CI [1.33; 10.68]) under desipramine. Milacipran revealed a RR of 1.06 (95%-CI [1.00; 1.13]) for overall adverse effects while overall adverse effects under treatment with venlafaxine had a RR of 1.44 (95%-CI [1.03; 2.02]) and 1.17 (95%-CI [1.06; 1.30]) under treatment with duloxetine. The reported RR for overall adverse effects under treatment with mirtazapine was 2.05 (95%-CI [0.54; 7.82]). Fluoxetine showed a RR of 3.78 (95%-CI [1.25; 11.43]) for overall adverse effects. Details are shown in Table 2 and Figures 2A–G. Comparative analysis of RDs for overall adverse effects of each drug showed that the placebo-adjusted risk to develop either side effect was highest under treatment with amitriptyline, mirtazapine, and fluoxetine. The lowest risk was under treatment with duloxetine and milnacipran (Figure 3).

**Table 2:** Risk ratio (RR) and corresponding 95%-confidence interval (CI) for overall adverse effects and withdrawal due to adverse effects.

Effect	Medication	Estimated RR	95%-CI	Strength of evidence
Withdrawal due to adverse effects				
	Amitriptyline	4.09	1.31; 12.82	**++**
	Nortriptyline	0.81	0.12; 5.44	**+−**
	**Desipramine**	**6.31**	**1.20; 33.14**	**++**
	**Milnacipran**	**2.28**	**1.87; 2.77**	**++**
	**Venlafaxine**	**3.10**	**1.15; 8.35**	**++**
	**Duloxetine**	**2.47**	**1.82; 3.36**	**++**
	Mirtazapine	5	0.25; 98.86	+
	Fluoxetine	1.81	0.2; 16.54	+
Overall adverse effects	Amitriptyline	2.9	0.67; 12.58	+
	Nortriptyline	1.5	0.17; 12.99	+
	**Desipramine**	**3.77**	**1.33; 10.68**	**++**
	Milnacipran	1.06	1.00; 1.13	0
	**Venlafaxine**	**1.44**	**1.03; 2.2**	**++**
	Duloxetine	1.17	1.06; 1.30	++
	Mirtazapine	2.05	0.54; 7.82	+
	**Fluoxetine**	**3.78**	**1.25; 11.43**	**++**

*Interpretation of RRs and 95%-CIs are shown as evidence: ++, strong evidence; +, intermediate evidence; +−, inconclusive results; 0, evidence of absence of a clinically relevant effect in either direction (limits for clinical relevance defined as 0.8 < RR < 1.2)*.

Strong evidence + statistical significance for AE/WDR for AD in “bold.”

#### Evidence for Withdrawals Related to Adverse Effects

**Figure 4:**
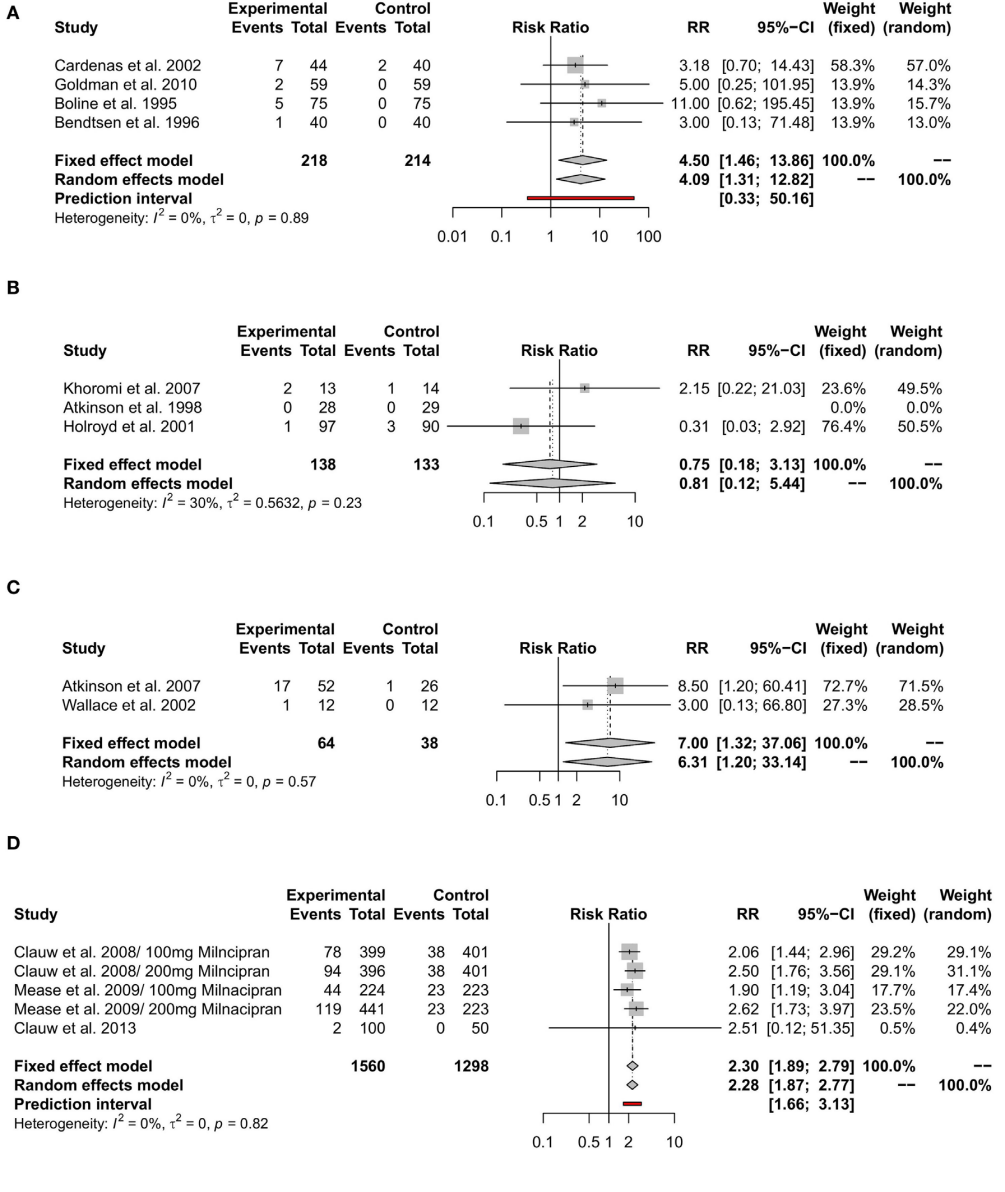

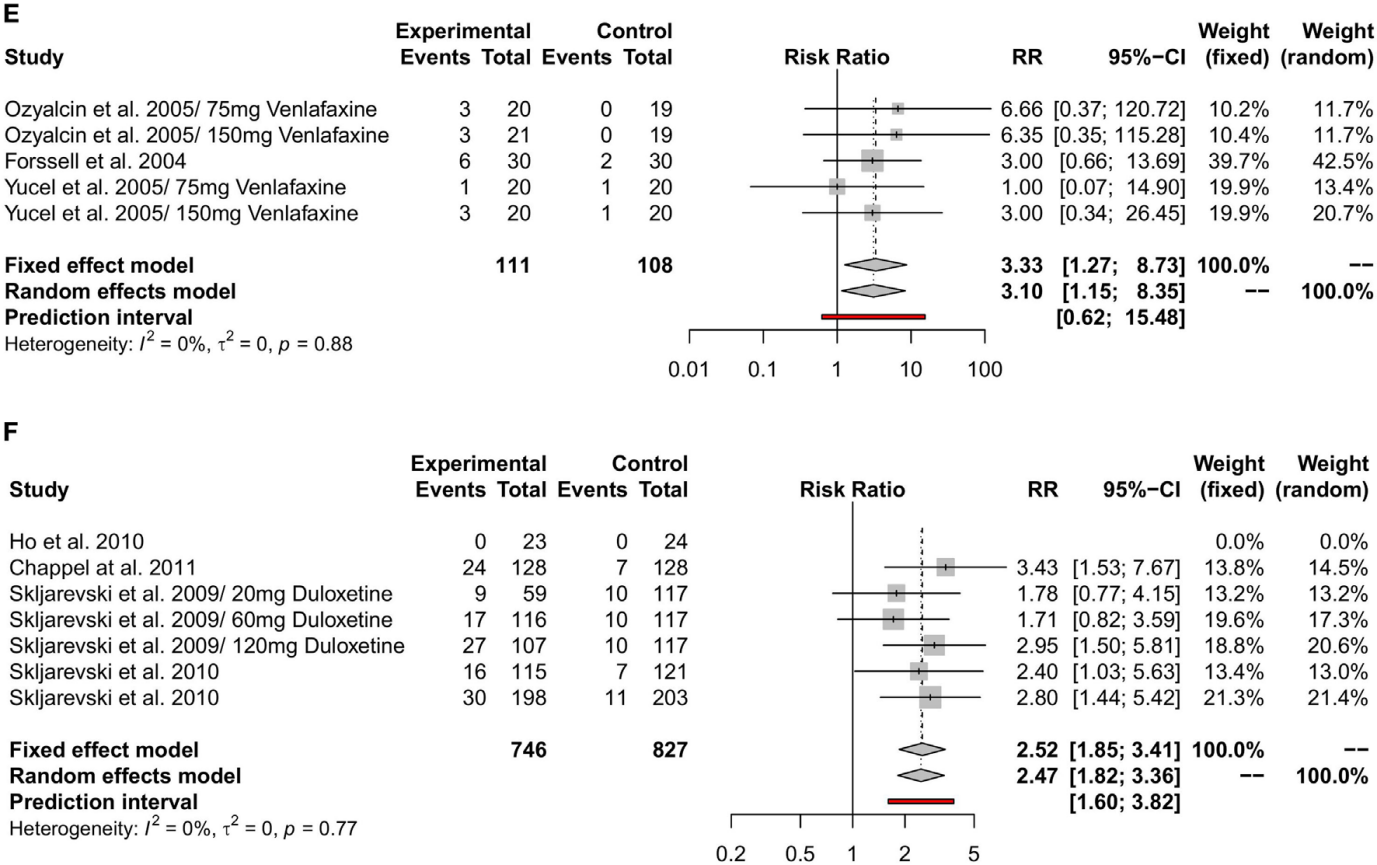
Meta-analyses of side effect-related withdrawal under treatment with different antidepressants. RR = risk ratio; 95%-CI = 95%-confidence interval; PI = prediction interval. Adverse effect-related withdrawal under treatment with amitriptyline **(A)**, with nortriptyline **(B)**, with desiparmine **(C)**, with milnacipran **(D)**, with venlafaxine **(E)**, and with duloxetine **(F)**.

Withdrawal due to adverse effects had a RR of 4.09 (95%-CI [1.31; 12.82]) under treatment with amitriptyline, 0.81(95%-CI [0.12; 5.44]) under treatment with nortriptyline, and 6.31 (95%-CI [1.20; 33.14]) under treatment with desipramine. The RR for treatment discontinuation due to adverse effects was 2.28 (95%-CI [1.87; 2.77]) for milnacipran, 3.10 (95%-CI [1.15; 8.35]) for venlafaxine, and 2.47 (95%-CI [1.82; 3.36]) for duloxetine. The reported RR for side effect-related withdrawal was 5 (95%-CI [0.25; 98.86]) for mirtazapine and 1.81 (95%-CI [0.2; 16.54]) for fluoxetine. Details are shown in Figures 4A–F.

**Figure 5:**
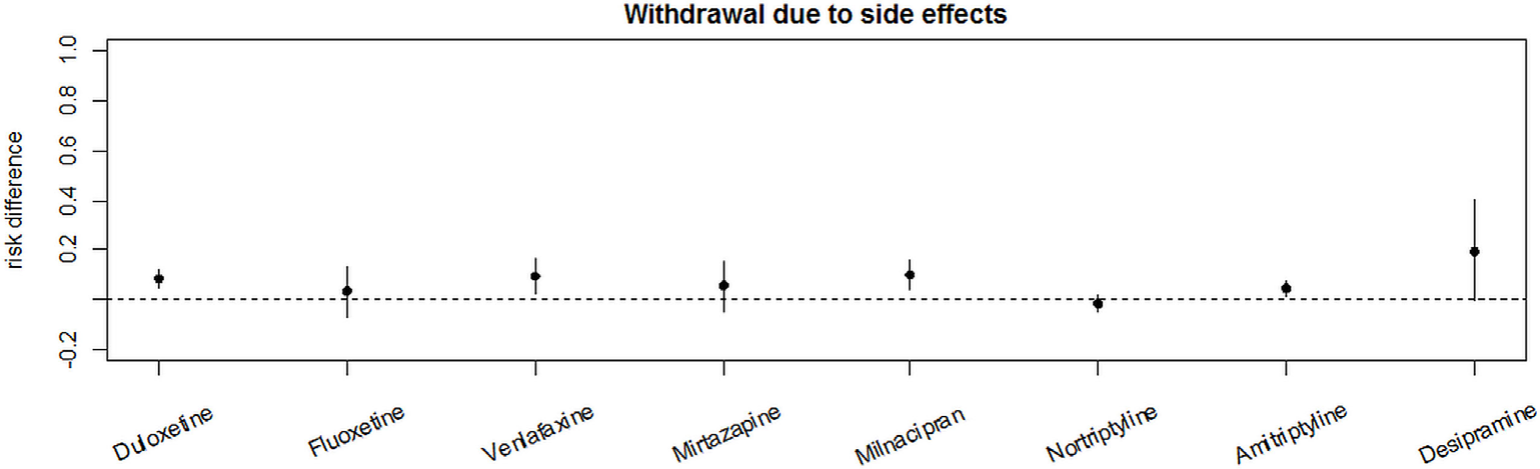
Comparative analysis of the placebo effect-adjusted risk for adverse effect-related withdrawal of the different analyzed antidepressants. Risk differences were used for placebo effect-adjusted risk.

Comparing RDs for side effect-related withdrawals shows the highest RD for desipramine, followed by duloxetine, venlafaxine, and milnaciprane. The lowest RD was seen in the nortriptyline group as shown in Figure 5.

#### Evidence for Specific Adverse Effects of Each Analyzed Antidepressant

##### Amitryptiline

Adverse effects with strong evidence *(*+++*/*++) of amitriptyline were dry mouth (RR 3.14; 95%-CI [1.10; 8.94]), headache (RR 3.39; 95%-CI [0.92; 12.55]), thirst (RR 18.95; 95%-CI [1.19; 301.39]), body weight gain (RR 8.74; 95%-CI [1.12; 68.32]), and constipation (RR 1.60; 95%-CI [1.19; 2.15]). Adverse effects with inconclusive evidence (±) of amitriptyline were gastritis, sweating, and dizziness under amitriptyline, headache and somnolence under nortriptyline. Adverse effects occurring more often in placebo groups (−−/−) of amitriptyline were insomnia and itching (strong evidence) and nausea, vomiting, urinating difficulties and abdominal pain (intermediate evidence).

##### Nortriptyline

Adverse effects with strong evidence (+++/++) of nortriptyline were constipation (RR 1.84; 95%-CI [0.90; 3.74]), drowsiness (RR 3.94; 95%-CI [2.14; 7.28]), and insomnia (RR 1.40; 95%-CI [0.92; 2.13]). Adverse effects with weak evidence (+) of nortriptyline were dizziness, dry mouth, heart burn, orthostatic hypotension, increased appetite, nervousness, palpitations, sedation, sweating, weight gain, and abdominal pain. Blurred vision is the only side effect occurring more frequently under placebo than under nortriptyline with intermediate evidence.

##### Desipramine

The side effect with strongest evidence (+++/++) under treatment with desipramine was dry mouth (RR 5.12; 95%-CI [1.41; 18.57]). Adverse effects with weak evidence (+) of desipramine were constipation, dizziness, drowsiness, headache, insomnia, loss of libido, nausea, shaking, sweating, and vomiting.

##### Milnacipran

Adverse effects with strong evidence (+++/++) under analgesic treatment with milnacipran were constipation (RR 4.52; 95%-CI [3.29; 6.20]), dizziness (RR 1.94; 95%-CI [1.46; 2.58]), dry mouth (RR 2.40; 95%-CI [1.27; 4.53]), headache (RR 1.62; 95%-CI [1.23; 2.12]), hot flush (RR 6.17; 95%-CI [3.42; 11.13]), hypertension (RR 3.31; 95%-CI [1.74; 6.28]), insomnia (RR 1.49; 95%-CI [0.98; 2.27]), nausea (RR 1.82; 95%-CI [1.60; 2.08]), palpitations (RR 3.46; 95%-CI [2.16; 5.53]), sweating (RR 4.97; 95%-CI [2.58; 9.57]), tachycardia (RR 5.56; 95%-CI [1.94; 15.94]), and vomiting (RR 2.77; 95%-CI [1.80; 4.26]). Adverse effects with inconclusive evidence (±) of milnacarpine were respiratory infections. Adverse effects occurring more often in placebo groups (−−/−) under placebo were diarrhea (strong evidence), edema, and nasopharyngitis (intermediate evidence).

##### Venlafaxine

Adverse effects with strong evidence (+++/++) of venlafaxine were drowsiness (RR 2.51; 95%-CI [0.86; 7.28]), somnolence (RR 7.39; 95%-CI [0.97; 56.50]), and vomiting (RR 16.07; 95%-CI [3.18; 81.29]). Adverse effects with weak evidence (+) of venlafaxine were constipation, jaw spasm, loss of libido, mydriasis, nausea, nightmares, sedation, sweating, urinating difficulty, headache, and fatigue. Adverse effects with inconclusive evidence (±) of venlafaxine were dizziness, dry mouth, loss of appetite, and palpitations.

##### Duloxetine

Adverse effects with strong evidence (+++/++) under duloxetine were constipation 4.02 (95%-CI [1.57; 10.33]), dry mouth 2.95 (95%-CI [1.17; 7.43]), hyperhidrosis 13.33 (95%-CI [1.76; 101.14]), and nausea 4.11 (95%-CI [1.15; 14.70]). Adverse effects with weak evidence (+) of duloxetine were dizziness, dyspnoe, hypertensive encephalopathy, muscular weakness, myocardial infarction, osteoarthritis, pruritus, and somnolence. Adverse effect with inconclusive evidence (±) of duloxetine was diarrhea. Adverse effects occurring more often in placebo groups (−−/−) under placebo were chest pain, headache, and vertigo.

##### Mirtazapine

Adverse effects with strong evidence (+++/++) under duloxetine were dizziness (RR 3.61; 95%-CI [0.96; 13.56]), drowsiness (RR 1.56; 95%-CI [0.84; 2.88]), and sedation (RR 3.5; 95%-CI [0.95; 12.9]). Adverse effects of weak evidence (+) under analgesic treatment with milnacipran were mostly arthralgia, fall, and fatigue. Adverse effects with weak evidence (+) for mirtazapine were concentration disturbances, dry mouth, edema, global weakness, metallic taste, swallowing difficulty, and weight gain. Under duloxetine remains unclear. Adverse effect with weak evidence (+) of mirtazapine was nausea under mirtazapine. Adverse effects occurring more often in placebo groups (−−/−) under placebo were fainting, irritability, increased appetite, and sleep disturbances (intermediate evidence).

##### Fluoextine

Fluoxetine showed adverse effects with strong evidence (Table 3). Adverse effects with weak evidence (+) of fluoxetine were breathlessness, dizziness, edema, headache, loss of appetite, loss of libido, nausea, and palpitations. Dry mouth was the only adverse effect that occurred more often under placebo than under fluoxetine (−−/−).

**Table 3:** Risk for specific adverse effects under treatment with the analyzed antidepressants.

Drug (prediction interval)	Side effect	Risk ratio	95%-CI	Evidence
**Amitriptyline**	**Dry mouth**	**3.14**	**1.10; 8.94**	**++**
	**Headache**	**3.39**	**0.92; 12.55**	**++**
	**Body weight gain**	**8.74**	**1.12; 68.32**	**++**
	**Thirst**	**18.95**	**1.19; 301.39**	**++**
	**Constipation**	**1.60**	**1.19; 2.15**	**++**
	Edema	1.8	0.18; 18.21	+
	Drowsiness	1.60	0.52; 4.91	+
	Irritability	8.19	0.45; 147.47	+
	Palpitations	1.55	0.29; 8.24	+
	Blurred vision	6.37	0.34; 119.6	+
	Diarrhea	1.21	0.29; 5.09	+
	*Gastritis*	*0.9*	*0.31; 2.61*	+−
	*Sweating*	*1.14*	*0.33; 3.94*	+−
	*Dizziness*	*1.12*	*0.47; 2.66*	+−
	Nausea	0.54	0.21; 1.38	−
	Vomiting	0.09	0; 1.59	−
	Abdominal pain	0.14	0.01; 2.68	−
	Urinating difficulty	0.40	0.04; 4.09	−
	**Insomnia**	**0.12**	**0.02; 0.94**	−−
	**Itching**	**0.42**	**0.16; 1.07**	−−
**Nortriptyline**	**Drowsiness**	**3.94**	**2.14; 7.28**	**++**
	**Constipation**	**1.84**	**0.90; 3.74**	**++**
	**Insomnia**	**1.40**	**0.92; 2.13**	**++**
	Dizziness	2.90	0.32; 26.10	+
	Dry mouth	2.04	0.75; 5.59	+
	Heart burn	3	0.14; 62.49	+
	Increase in appetite	2.78	0.11; 67.49	+
	Nervousness	2.78	0.11; 0.67	+
	Orthostatic hypotension	1.26	0.78; 2.03	+
	Palpitations	1.55	0.28; 8.61	+
	Sedation	1.26	0.78; 2.03	+
	Sweating	1.40	0.62; 3.15	+
	Weight gain	2.90	0.32; 26.10	+
	Abdominal pain	2.78	0.11; 67.49	+
	*Somnolence*	*1*	*0.08; 13.02*	+−
	*Headache*	*1*	*0.08; 13.02*	+−
	Blurred vision	0.33	0.02; 6.94	−
**Desipramine**	**Dry mouth**	**5.12**	**1.41; 18.57**	**++**
	Constipation	5.78	0.74; 44.97	+
	Dizziness	3	0.13; 66.8	+
	Drowsiness	11	0.68; 178.53	+
	Headache	5	0.27; 93.96	+
	Insomnia	5	0.27; 93.96	+
	Loss of libido	2.21	0.09; 51.85	+
	Nausea	5	0.27; 93.96	+
	Shaking	5	0.27; 93.96	+
	Sweating	7	0.4; 121.94	+
	Vomiting	3	0.13; 66.8	+
**Milnacipran**				
**2.26; 9.04**	**Constipation**	**4.52**	**3.29; 6.20**	**+++**
**1.37; 5.57**	**Vomiting**	**2.77**	**1.80; 4.26**	**+++**
**1.04; 3.62**	**Dizziness**	**1.94**	**1.46; 2.58**	**+++**
**1.47; 2.25**	**Nausea**	**1.82**	**1.60; 2.08**	**+++**
**1.04; 2.50**	**Headache**	**1.62**	**1.23; 2.12**	**+++**
**1.09; 10.98**	**Palpitations**	**3.46**	**2.16; 5.53**	**+++**
	**Dry mouth**	**2.40**	**1.27; 4.53**	**++**
	**Hot flush**	**6.17**	**3.42; 11.13**	**++**
	**Hypertension**	**3.31**	**1.74; 6.28**	**++**
	**Insomnia**	**1.49**	**0.98; 2.27**	**++**
	**Sweating**	**4.97**	**2.58; 9.57**	**++**
	**Tachycardia**	**5.56**	**1.94; 15.94**	**++**
	Arthralgia	1.5	0.16; 14.06	+
	Fall	4.52	0.25; 82.36	+
	Fatigue	2	0.23; 17.43	+
	*Respiratory infections*	*1.07*	*0.71; 1.62*	+−
	Edema	0.33	0.06; 1.93	−
	Nasopharyngitis	0.73	0.48; 1.12	−
	Diarrhea	0.68	0.42; 1.11	−−
**Venlafaxine**	**Vomiting**	**16.07**	**3.18; 81.29**	**++**
	**Somnolence**	**7.39**	**0.97; 56.50**	**++**
	**Drowsiness**	**2.51**	**0.86; 7.28**	**++**
	Constipation	1.76	0.51; 6.03	+
	Jaw spasm	4.39	0.52; 36.98	+
	Loss of libido	4.39	0.52; 36.98	+
	Mydriasis	3.64	0.42; 31.56	+
	Nausea	3.13	0.74; 13.85	+
	Nightmares	1.22	0.68; 2.21	+
	Sedation	6.35	0.35; 115.28	+
	Sweating	1.98	0.18; 21.37	+
	Urinating difficulty	1.5	0.67; 3.34	+
	Headache	1.06	0.95; 1.18	0
	Fatigue	1.06	0.95; 1.18	0
	*Dizziness*	*0.93*	*0.25; 3.45*	+−
	*Dry mouth*	*1.04*	*0.66; 1.64*	+−
	*Palpitations*	*1.08*	*0.74; 1.57*	+−
	*Loss of appetite*	*1.08*	*0.79; 1.47*	+−
**Duloxetine**	**Constipation**	**4.02**	**1.57; 10.33**	**++**
	**Dry mouth**	**2.95**	**1.17; 7.43**	**++**
	**Hyperhidrosis**	**13.33**	**1.76; 101.14**	**++**
	**Nausea**	**4.11**	**1.15; 14.70**	**++**
	Dizziness	2.72	0.77; 9.57	+
	Dyspnoe	3.03	0.12; 73.52	+
	Hypertensive encephalopathy	3.16	0.13; 76.69	+
	Muscular weakness	3.03	0.12; 73.52	+
	Myocardial infarction	1.52	0.24; 9.61	+
	Osteoarthritis	3.16	0.13; 76.69	+
	Pruritus	3.13	0.13; 73	+
	Somolence	2.47	0.24; 25.39	+
	TIA	3.22	0.34; 30.70	+
	Wrist fracture	3.16	0.13; 76.69	+
	*Diarrhea*	*1.19*	*0.42; 3.37*	+−
	Headache	0.73	0.20; 2.59	−
	Vertigo	0.70	0.14; 3.47	−
	Chest pain	0.70	0.12; 4.02	−
**Mirtazapine**	**Dizziness**	**3.61**	**0.96; 13.56**	**++**
	**Drowsiness**	**1.56**	**0.84; 2.88**	**++**
	**Sedation**	**3.5**	**0.95; 12.9**	**++**
	Concentration disturbances	2	0.19; 20.61	+
	Dry mouth	1.85	0.43; 7.94	+
	Edema	1.5	0.27; 8.19	+
	Global weakness	3	0.14; 65.55	+
	Metallic taste	3	0.14; 65.55	+
	Swallowing difficulty	3	0.14; 65.55	+
	Weight gain	6	0.78; 46.14	+
	*Nausea*	*1*	*0.15; 6.53*	+−
	Sleep disturbances	0.67	0.12; 3.64	−
	Irritability	0.2	0.03;1.59	−
	Increased appetite	0.6	0.16; 2.23	−
	Fainting	0.33	0.02; 7.28	−
**Fluoxetine**	Breathlessness	2.58	0.11; 59.62	
	Dizziness	1.71	0.17; 17.38	+
	Edema	1.71	0.17; 17.38	+
	Headache	1.29	0.24; 6.86	
	Loss of appetite	2.58	0.11; 59.62	
	Loss of libido	10.71	0.64; 178.16	
	Nausea	1.42	0.4; 5.17	
	Palpitations	2.58	0.11; 59.62	
	*Gastritis*	*1.2*	*0.46; 3.13*	+−
	Dry mouth	0.35	0.03; 3.67	−

*Evidence is estimated according to risk ratios and corresponding 95%-CI obtained from meta-analyses*.

*+++/++, strong evidence for side effect under treatment; +, weak evidence for side effect under treatment; +−, inconclusive evidence; −, weak evidence for placebo-induced side effect; −−, strong evidence for placebo-induced side effect; RR, risk ratio; 95%-CI, 95%-confidence interval; PI, prediction interval*.

Symptoms in “bold” show statistical significance + strong evidence, underlined symptoms are more often under placebo than under therapy, and evidence remains unclear for symptoms in “italic.”

Risks for specific adverse effects under treatment with the analyzed antidepressants are detailed in Table 3.

#### Comparative Analysis of RDs of Each Drug for the Most Common Adverse Effects

**Figure 6:**
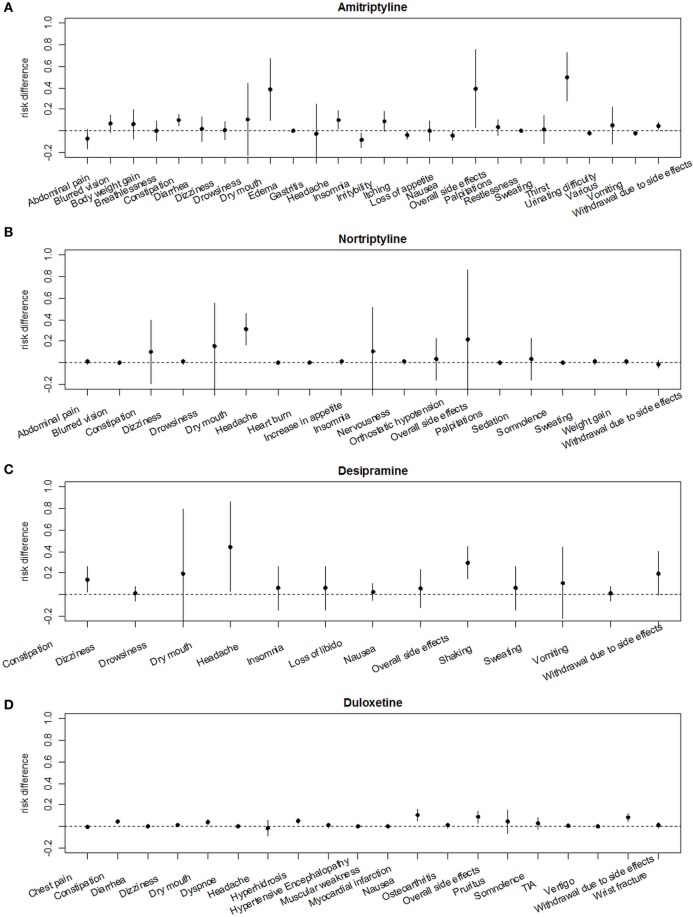

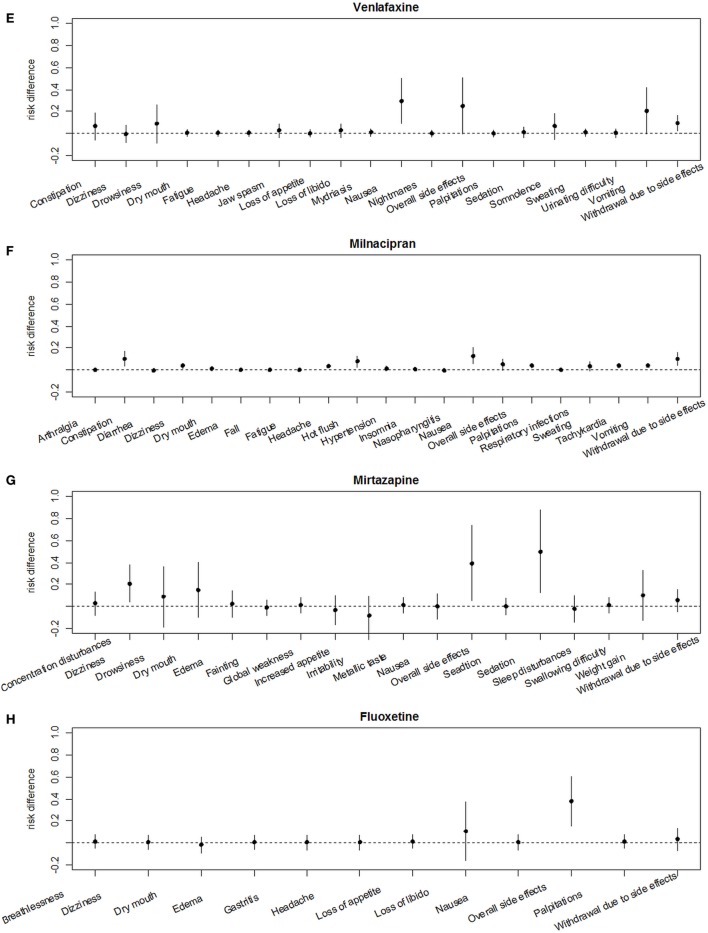
Comparative analyses of adverse effects of each analyzed drug using risk differences. **(A)** Amitriptyline, **(B)** nortriptyline, **(C)** desipramine, **(D)** duloxetine, **(E)** venlafaxine, **(F)** milnacipran, **(G)** mirtazapine, **(H)** fluoxetine.

Risk differences for each adverse effect are illustrated in (Figures 6A–H). Regarding all analyzed drugs in this meta-analysis the most common adverse effects were dry mouth, dizziness, followed by nausea, headache, constipation, sweating/hyperhidrosis, drowsiness, and palpitations.

Comparative analyses of placebo-adjusted RDs revealed the highest risk to develop “dry mouth” for desipramine, amitriptyline, nortriptyline, and mirtazapine (Figure S1 in Supplementary Material), while the highest risk for dizziness was seen under mirtazapine (Figure S1B in Supplementary Material). Risk of nausea occurred was highest under venlafaxine, duloxetine, and milnacipran, and was low under amitriptyline (Figure S1C in Supplementary Material). Risk of headache was higher under treatment with amitriptyline and desipramine, while risk of constipation was almost equal for TCAs and SSRIs (Figures S1C,D in Supplementary Material). Risk of drowsiness was most severe under therapy with despramine and nortriptyline, followed by amitriptyline, mirtazapine, and venlafaxine. Risk of hyperhidrosis was most pronounced under desipramine, duloxetine, and milnacipran. Risk of palpitations was highest under amitriptyline and milnacipran (Figures S1F–H in Supplementary Material).

## Discussion

The major findings of our analyses are (1) all antidepressants included in the analysis, except nortriptyline, showed higher incidence of adverse effects compared to placebo, (2) amitriptyline, mirtazapine, desipramine, venlafaxine, fluoxetine, and nortriptyline showed the highest placebo effect-adjusted risk of adverse effects, (3) risk for withdrawal due to side effects was highest in desipramine, milnacipran, venlafaxine, and duloxetine, (4) the most frequent adverse effects under treatment with antidepressants were dry mouth, dizziness, nausea, headache, and constipation followed by palpitations, sweating, and drowsiness, and (5) antidepressants showed specific adverse effect profiles. Taken together, our synthesized data confirm previous studies demonstrating safety of antidepressants in the treatment of chronic pain and further advance these results. Distinct profiles of adverse effects identified in this analysis might provide useful information for personalized multimodal treatment which takes both comorbidities and co-medication into consideration.

Adverse effects due to antidepressive treatment have heterogeneous mechanisms of action. TCAs, for example, block histaminic, cholinergic, and alpha1-adrenergic receptor sites, resulting in occurrence of adverse effects, including weight gain, dry mouth, constipation, drowsiness, and dizziness (43). Monoamine oxidase inhibitors show an interaction with tyramine which can lead to severe arterial hypertension and furthermore show interactions with numerous drugs. The newer generation of antidepressants, SSRIs are single-receptor selective drugs which target one specific brain receptor site without agonizing unwanted receptor sites or transmitters, such as histamine and acetylcholine. Importantly, in the majority of cases those adverse effects usually appear with initiation of treatment while therapeutic benefits may be delayed. However, in our analyses, differences in the delay of onset of analgetic action may reduce comparibility of studies, particularly those with shorter durations of observation.

TCA is the first group of antidepressants used in the treatment of pain with decades of clinical experience. Treatment costs are comparably low (43). Previous prospective studies in patients with chronic pain highlighted adverse effects, such as blurred vision, urinary retention, constipation, and dry mouth, as well as antihistaminic adverse effects, such as weight gain and sedation. Our synthesized analysis not only confirmed strong evidence for dry mouth, thirst, constipation, headache, and gain in body weight but also blurred vision and palpitations. Adjusting results for potential placebo effects seems to be particularly relevant as insomnia and itching occurred as important side effect under placebo as well as nausea, vomiting, abdominal pain, and urinating difficulty. In placebo-controlled studies of amitriptyline, our synthesized analyses also showed a significant placebo related risk for these adverse effects observed might point to a specific protective effect of amitriptyline or might be explained by the complex underlying pathophysiology of chronic pain forms.

Similar to our results, Finnerup et al. reported somnolence, constipation, and dry mouth as major adverse effects for TCA in a recent review and meta-analysis of studies of neuropathic pain management (44). As opposed to studies of TCA in patients with depression, severe adverse effects related to hypotension or prolonged QT intervals were not evident in our analyses (45). This might be due to the fact that lower doses were necessary in analgesic treatment compared to those doses that are needed for treatment for depression. More recently designed antidepressants such as SSNRI were suggested to cause fewer adverse effects than TCA while yielding similar efficacy in the treatment of depression (46). The mechanism of action of SSNRI is a dual inhibition of 5HT and NE reuptake. However, selectivitiy and intenstity of inhibition differ among SSNRIS, probably explaining diffrences in the adverse effect profiles. Duloxetine and venlafaxine are more selective for serotonin reuptake inhibition at lower doses compared to milnacipran. Milnacipran shows higher selectivity for norepinephrine compared to duloxetine and venlafaxine (21–32). Adverse effects occurring with highest evidence under treatment with venlafaxine are vomiting, somnolence, and drowsiness. RD added nausea and constipation as important adverse effects. Neither palpitations nor hypertension was observed. This might be related to the fact that venlafaxine was applied in sub-antidepressive doses, where the threshold for noradrenergic adverse effects might not have been reached. However, in an experimental setting, venlafaxine led to complex disturbances of the autonomic nervous system in a dose-dependent fashion even in the lower doses, possibly indicating a continuous dose-dependent rather than a threshold-based all or nothing mechanism of noradrenergic adverse effects (47, 48). Cognitive functions were not altered in the same expiremental setting, consistent with a predominant effect on centers of autonomic sympathetic control and efferent adrenergic pathways (49). Even though acting in a similar fashion, different adverse effects were observed for duloxetine. Our synthesized analyses revealed that constipation, dry mouth, hyperhidrosis, and nausea are adverse effects of venlafaxine with strong evidence. Compared to a previous analysis that has identified only nausea as adverse effect with the same level of evidence, our analyses suggest a less favorable side effect profile of venlafaxine (44). In fact, our analyses indicate that, among assessed antidepressants, milnacipran shows the highest degree of evidence for several specific adverse effects including constipation, vomiting, dizziness, nausea, headache and palpitations, dry mouth, hot flush, hypertension, insomnia, sweating, and tachycardia. However, using RD for placebo-adjusted risks, only constipation, nausea, hot flush, palpitations, tachycardia, and vomiting emerged as high risk adverse effects. Interestingly, our analyses showed that diarrhea occurred less often under milnacipran compared to placebo. As constipation is a side effect occurring with strong evidence under milnacipran, a protective effect of milnacipran against diarrhea appears plausible. This might be relevant to the treatment of patients with chronic pain and gastroenterologic comorbidities.

Our meta-analysis revealed that under treatment with mirtazapine dizziness, drowsiness, and sedation are important adverse effects. However, RD that adjusted adverse effects for placebo identified sedation, weight gain, dizziness, drowsiness, dry mouth, and concentration disturbances as main adverse effects, possibly indicating a stronger clinically relevant anticholonergic component to the underlying mechanism of action. Consistently, irritability, increased appetite and fainting occurred less often under treatment with mirtazapine compared to placebo, possibly due to a protective effect of the drug.

While adverse effects such as gastrointestinal symptoms, nausea, vomiting, diarrhea, and sexual dysfunction are frequently reported under treatment with fluoxetine for depression, the present analysis of fluoxetine revealed no adverse effect occurring with strong evidence (50, 51). However, RD-based analyses showed nausea and palpitations to occur with high evidence. This profile is consistent with the absence of any anticholinergic action and confirms previous research indicating a beneficial adverse effect profile of SSRI compared to TCA (46). However, SSRI are less effective for analgesic treatment which might be due to a significant noradrenergic component to the mechanism whereby antidepressants alleviate pain.

The highest evidence for overall adverse effects was seen under treatment with desipramine, venlafaxine, and fluoxetine by analyzing RRs However, the drugs most likely leading to discontinuation of the medication were desipramine, milnacipran, venlafaxine, and duloxetine possibly indicating that severity and unpleasant perception of adverse effects was more pronounced under treatment with these antidepressants compared to, e.g., the SSRI fluoxetine and milnacarpine.

Overall, adverse effect rate under treatment with placebo was relatively high possibly reflecting increased awareness and expectancy of adverse effects in patients with chronic pain. The induction or the worsening of symptoms induced by placebo administration also referred to as “nocebo effect” might particularly be relevant in those patients that suffer from long-term illness with unpleasant symptoms such as chronic pain. It is important to point out that, compared to other analgesic drugs, adverse effects of antidepressants have been shown reversible and not linked to any structural organ damage (52).

There are recommendations for symptomatic treatment of underlying specific diseases of chronic pain. Some recommendations are approved by the FDA, but often individual treatment regimens and off-label use are applied. Fibromyalgia syndrome is defined as diffuse pain for more than 3 months and at least 11 out of 18 defined tender points by the American College of Rhematology, fatigue, sleep disturbances, depression, and cognitive dysfunction (53). The mechanism of pain is unclear, but one explanation might be the enhanced central sensitization. In addition, psychological factors, such as depression, anxiety, and stress, contribute to chronification of pain in these patients. Besides non-pharmacological treatments, TCA and SSRI are recommended. It has been shown that treatment with fluoxetine is less effective than TCA in the treatment of fibromyalgia related chronic pain. However, the clinical decision on whether selecting a TCA or SSRI for pain treatment in these patients should also take into account specific profiles of adverse effects. Patients with fibromyalgia suffer frequently from limited functional status (54). This symptom might be further deteriorated by drowsiness, an adverse effect for which our analysis showed the highest risk in despramine and nortriptyline. Duloxetine and milnacipran are also approved by the FDA for treatment of fibromyalgia (55–57).

Neuropathic pain occurs in up to 50% of patients with peripheral neuropathy (58). The major etiology for neuropathic pain is diabetic neuropathy. 21% of patients suffering from diabetes mellitus type 2 more than 10 years develop diabetic neuropathy. Effectiveness in treatment of diabetic neuropathy has been shown for TCAs as amitriptyline, clomipramine, and imipramine as well as for SSRIs as fluoxetine, citalopram, and paroxetine. However, most studies show higher effectiveness for TCAs—amitriptyline, nortriptyline, desipramine, and imipramine—compared to SSRIs paroxetine and fluoxetine (59–62). Duloxetine was approved by FDA for the treatment of diabetic neuropathy at doses of 60 mg/day. Even though not approved, venlafaxine has shown positive effects in off-label use at doses of 150–225 mg/day. Patients with diabetic neuropathic frequently suffer from complex disturbances of the autonomic nervous system with cardiovascular, gastroenterologic and urogenital, and other symptoms which is why an accurate matching between risk for adverse effects of the selected antidepressant and manifest autonomic symptoms appears beneficial (63). For instance, nausea is a prevalent symptom of diabetic neuropathy which showed highest risk of occurrence in our analysis under treatement with amitriptyline (64). While this does not prove a clinically relevant additive effect of TCA treatment and neuropathy in the development of nausea, knowledge of this observation might prove useful in the multimodal treatment of diabetic neuropathy.

Postherpetic neuralgia results after infection with varicella zoster virus, a neurotropic virus that can remain hidden in ganglion of sensory cranial nerves and can be reactivated as late onset even years after infection as acute herpes zoster (65). Postherpetic neuralgia is a ususally drug resistant pain that lasts longer than 3 months in the skin area formerly affected by herpes zoster (66–68). Current guidelines recommend therapy with TCA, tramadol or opioids while TCA and alpha2 ligands are the most common drugs (69). Effectiveness has been shown for treatment with TCA—namely amitriptyline, desipramine, and nortriptyline, as well as for treatment with SSRI as fluoxetine. Since pain is usually severe and can last for years, causing physical and social disability focus of treatment should be predominantly driven by efficacy in pain reduction (70). However, adverse effects should be anticipated, which might be particularly relevant when headache is induced by the drug, an adverse effect which showed highest risk under treatment with amitriptyline in our analysis. Chronic low-back pain is backache that lasts for at least 3 months often associated with radiculopathy or lumbar canal stenosis (71, 72) and is persistent in up to 45% of affected patients. Assumed pathophysiological mechanisms include changes of the central nervous system with neuronal hyperactivity, membrane excitability, and consequent dysfunction of inhibitory systems. Short-term efficacy of NSAIDs and opioids has been shown, but chronic pain is commonly treated by antidepressants (73). TCA are effective in alleviating chronic low-back pain but show significant adverse effects (74, 75). SNRIs show lower efficacy and less adverse effects in the treatment of chronic low-back pain (76–78). Duloxetine is activating descending inhibitory pathways in the brain stem and the spinal cord which might explain its efficacy in the treatment of chronic low-back pain (29). While efficacy in pain reduction is the primary goal of treatment, complex effects of chronic low-back pain on biologic, psychologic, and social aspects need to be considered when selecting an antidepressive treatment as part of multimodal therapy (79). Adverse effects such as dizziness and drowsiness, which showed highest risk under mirtazapine; despramine and nortriptyline, respectively, in our analysis, might further contribute to already impaired functional status and increased risk of falls in elder patients with chronic low-back pain (80). This might be similarly relevant to patients with osteoarthritis induced chronic pain which is also more prevalent in the elderly (81).

Standard treatment for chronic tension-type headache is amitriptyline (82). Amitriptyline is the only drug with prophylactic effect, but the mechanism is unknown. SSRI have no or limited analgesic effect and it has been shown that SSRI are no more effective than placebo. Venlafaxine is efficacious for chronic tension-type headache. Efficacy of mirtazapine has been shown to be comparable to amitriptyline. Recommended treatments for chronic tension-type headache are amitriptyline, clomipramine, maprotiline and mirtazapine while migraine should be treated by amitriptyline, fluoxetine and venlafaxine. While our analysis showed that among antidepressants risk of headache was highest under treatment with amitriptyline an additive mechanism of this adverse effect in chronic tension-type headache appears not plausible when viewed in conjunction with evidence of efficacy in reducing headache in these patients.

Several recommendations for the treatment of specific chronic pain states are available (83–89). Recommendations for the treatment of chronic pain comprise TCA, pregabalin, gabapentin, and lidocaine as first-line treatment (84, 85, 89–91). In contrast; a systemic review and meta-analysis applied a standardized grading system (GRADE Grading of Recommendations Assessments, Development, and Evaluation) to avoid bias and create evidence-based recommendations and provided different recommendations (8, 44, 92). Recommendations for treatment of chronic neuropathic pain comprised duloxetine and venlafaxine, TCA as well as pregabalin and gabapentine as first-line therapy. Recommendations for venlafaxine and duloxetine were of high evidence, while recommendations for TCA were of moderate evidence. Interestingly, in the present meta-analysis venlafaxine was one of the substances most likely to induce adverse effects with high risk for the need of withdrawal. In contrast; nortriptyline showed a better safety profile. No clear superiority of one substance over another has been shown in our analysis; selection of the specific drug should rather be guided by specific adverse effect profiles in a personalized approach which also takes into account the patients’ individual comorbidities and health status. Patients with chronic pain who have to drive or have to operate machinery due to their profession should preferably not be treated with antidepressants leading mainly to drowsiness, dizziness and somnolence such as mirtazapine, venlafaxine, nortriptyline, amitriptyline and desipramine. For those patients, drugs as milnacipran and duloxetine might be more beneficial. By contrast, patients suffering from sleep disturbances in addition to chronic pain might benefit from drugs with these adverse effects. However, mirtazapine is beneficial of sleep at 7.5 and 15 mg only. Dry mouth might be particularly unpleasant in patients who need to speak a lot. Those patients might benefit more from antidepressants with low incidence of dry mouth such as venlafaxine and milnacipran. Some antidepressants can lead to cardiovascular adverse effects such as prolongation of QT interval, hypertension or arrhythmias. Although evidence on these adverse effects was low in our analysis, tachycardia and palpitations occurred mostly under treatment with milnacipran, amitriptyline and fluoxetine. Consequently, these effects should be taken into consideration in patients with high cardiovascular risk.

Disturbance of sexual function or urinating difficulties reduce quality of life (93). In the present analysis, these adverse effects showed comparably low incidence for the analyzed drugs. This might be related to the fact that patients report infrequently about these symptoms to avoid embarrassing situations or might be due to the lower doses of antidepressants applied in included studies compared to antidepressive dosages. In the present analysis, disturbance of sexual function or urinating difficulties, adverse effects were observed under duloxetine, desipramine, and fluoxetine.

While further elaboration of the findings from our pooled analyses is warranted to optimize individualized pharmacotherapy in patients who are suffering from chronic pain, prospective preventive research is equally needed. In fact, specific guidelines exist for the conduction of prevention clinical trials in this population that pose a useful guide to researchers (94).

## Conclusion

Based on the meta-analytic comparison of adverse effect rates between antidepressants and placebo our study confirm tolerability of low-dose antidepressants for the treatment of chronic pain and reveals specific profiles of adverse effects that differ from those of higher doses of the same drugs applied for depression. These findings might be useful in multimodal treatment which takes patient comorbidities and co-medication into consideration. Pathophysiology of the underlying disease, comorbidities, lifestyle, and co-medication should be taken into consideration when determining the use of an antidepressant in patients with chronic pain.

## Author Contributions

CR has made substantial contributions to analysis and interpretation of the data. She wrote the first draft of the manuscript. TS, KB, SM, and JW have made substantial contributions to interpretation of the data and reviewing the manuscruipt for intellectual content. TS has made substantial contributions to design of the analyses, interpretation, and revising the manuscript for intellectual content. He is the principal researcher and corresponding author.

## Conflict of Interest Statement

The authors declare that the research was conducted in the absence of any commercial or financial relationships that could be construed as a potential conflict of interest.
