# Supplementary material for: Adverse Effects of Antidepressants for Chronic Pain: A Systematic Review and Meta-analysis
Source: Front Neurol. 2017 Jul 14;8:307. doi: 10.3389/fneur.2017.00307 (PMC5510574; doi:10.3389/fneur.2017.00307)

**Supplementary data**

**Appendices**

**Appendix S App-1**

Pain conditions included in the analyses were chronic low back pain, fibromyalgia, rheumatic pain, migraine, chronic tension-type headache, atypical facial pain, chronic arm pain, neuropathic pain, chronic low back pain, capsaicin- induced pain in healthy participants, post mastectomy pain, pain after knee replacement surgery and knee osteoarthritis pain

**Supplementary tables**

**Table S-1 Subgroups of antidepressants. Drugs analyzed in our analyses are marked with blue color.**

| **TCA** | | | **Selective reuptake inhibitors** | | | | **Noradren-ergic and specific seroton-ergic anti-depressants**  **(NaSSA)** | **Serotonin-antagonist and reuptake inhibitor**  **(SARI)** |
| --- | --- | --- | --- | --- | --- | --- | --- | --- |
| Non-selective Monoamino-reuptake inhibitors | | |  | | | |
| Serotonin-reuptake inhibitors (SRI) | Noradrenaline-reuptake inhibitors (NRI) | Serotonie-Noradrenalin reuptake inhibitors (SNRI) | Selective Serotonin reuptake inhibitors (SSRI) | Selective noradrenaline reuptake inhibitors (SNRI) | Selective Serotonine-noradrenaline reuptake inhibitors (SSNRI) | Selective noradrenaline-dopamin reuptake inhibitors (SNDRI) |
| Clomi-pramine | Desipramine | Amitriptyline | Fluoxetine | Reboxe-tine | Duloxetine | Bupropion | Mirtazapine | Trazo-done |
|  | Nortriptyline | Imipramine | Fluvox-amine |  | Venlafaxine |  | Mianserin |  |
|  |  | Doxepin | Citalopram |  | Milnacipran |  |  |  |
|  |  | Dibenzepin | Es-citalopram |  |  |  |  |  |
|  |  |  | Sertraline |  |  |  |  |  |
|  |  |  | Paroxetine |  |  |  |  |  |

**Table S-2:** Adverse effects occurring under analgesic treatment with antidepressants.

| **Adverse effects** | **Amitriptyline** | **Nortriptyline** | **Desipramine** | **Duloxetine** | **Venlafaxine** | **Milnacipran** | **Fluoxetine** | **Mirtazapine** |
| --- | --- | --- | --- | --- | --- | --- | --- | --- |
| Nausea |  |  |  |  |  |  |  |  |
| Drowsiness |  |  |  |  |  |  |  |  |
| Dizziness |  |  |  |  |  |  |  |  |
| Vomiting |  |  |  |  |  |  |  |  |
| Dry mouth |  |  |  |  |  |  |  |  |
| Constipation |  |  |  |  |  |  |  |  |
| Headache |  |  |  |  |  |  |  |  |
| Somnolence |  |  |  |  |  |  |  |  |
| Sedation |  |  |  |  |  |  |  |  |
| Insomnia |  |  |  |  |  |  |  |  |
| Gain in body weight |  |  |  |  |  |  |  |  |
| Increase in appetite |  |  |  |  |  |  |  |  |
| Loss of appetite |  |  |  |  |  |  |  |  |
| Peripheral edema |  |  |  |  |  |  |  |  |
| Hypertension |  |  |  |  |  |  |  |  |
| Tachykardia |  |  |  |  |  |  |  |  |
| Palpitations |  |  |  |  |  |  |  |  |
| Hot flush |  |  |  |  |  |  |  |  |
| Sweating |  |  |  |  |  |  |  |  |
| Loss of libido |  |  |  |  |  |  |  |  |
| Ejaculation disorder |  |  |  |  |  |  |  |  |
| Urinating difficulty |  |  |  |  |  |  |  |  |
| Gastritis |  |  |  |  |  |  |  |  |
| Diarrhea |  |  |  |  |  |  |  |  |
| Abdominal pain |  |  |  |  |  |  |  |  |
| Vertigo |  |  |  |  |  |  |  |  |
| Unsteadiness |  |  |  |  |  |  |  |  |
| Increased sleep |  |  |  |  |  |  |  |  |
| *Itching* |  |  |  |  |  |  |  |  |
| Thirst |  |  |  |  |  |  |  |  |
| Irritability |  |  |  |  |  |  |  |  |
| Blurred vision |  |  |  |  |  |  |  |  |
| Heart burn |  |  |  |  |  |  |  |  |
| Arthralgia |  |  |  |  |  |  |  |  |
| Orthostatic hypotension |  |  |  |  |  |  |  |  |
| Cough |  |  |  |  |  |  |  |  |
| *AP* |  |  |  |  |  |  |  |  |
| *ASAT* |  |  |  |  |  |  |  |  |
| *GGT* |  |  |  |  |  |  |  |  |
| *Decrease of body weight* |  |  |  |  |  |  |  |  |
| Shaking |  |  |  |  |  |  |  |  |
| *Malaise* |  |  |  |  |  |  |  |  |
| Nasopharyngitis |  |  |  |  |  |  |  |  |
| Respiratory infections |  |  |  |  |  |  |  |  |
| Pain in extremity |  |  |  |  |  |  |  |  |
| Fall |  |  |  |  |  |  |  |  |
| *Influenza* |  |  |  |  |  |  |  |  |
| *Hyppothyreodism* |  |  |  |  |  |  |  |  |
| *Neck pain* |  |  |  |  |  |  |  |  |
| Hypertension |  |  |  |  |  |  |  |  |
| Metallica Taste |  |  |  |  |  |  |  |  |
| Weakness |  |  |  |  |  |  |  |  |
| Difficuty in swallowing |  |  |  |  |  |  |  |  |
| Mydriasis |  |  |  |  |  |  |  |  |
| Jaw spasm |  |  |  |  |  |  |  |  |
| Breathlessness |  |  |  |  |  |  |  |  |
| Anxiety |  |  |  |  |  |  |  |  |
| Sleep disturbances |  |  |  |  |  |  |  |  |
| Nightmares |  |  |  |  |  |  |  |  |
| Fatigue |  |  |  |  |  |  |  |  |
| Difficulties in concentration |  |  |  |  |  |  |  |  |
| TIA |  |  |  |  |  |  |  |  |
| Myocardial infarction |  |  |  |  |  |  |  |  |

Table of side effects per antidepressant drug. Light colors refer to mild to moderate side effects, dark colors indicate severe side effects.

**Supplementary figures**

**Figure S-1:** Placebo effect- adjusted risk using risk differences for the most common adverse effects occurring under the analyzed antidepressants.

**Figure S-1 a:** Placebo effect- adjusted risk using risk differences for dry mouth


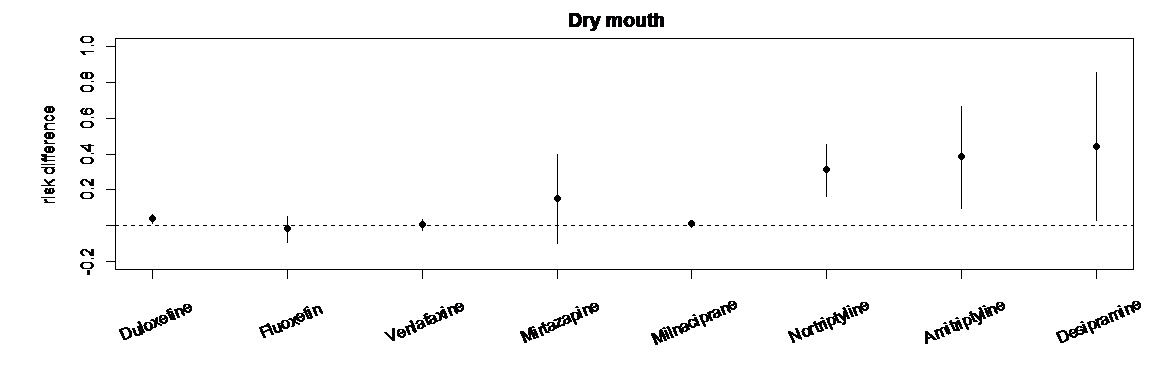


**Figure S-1 a:** Placebo effect- adjusted risk using risk differences for dizziness


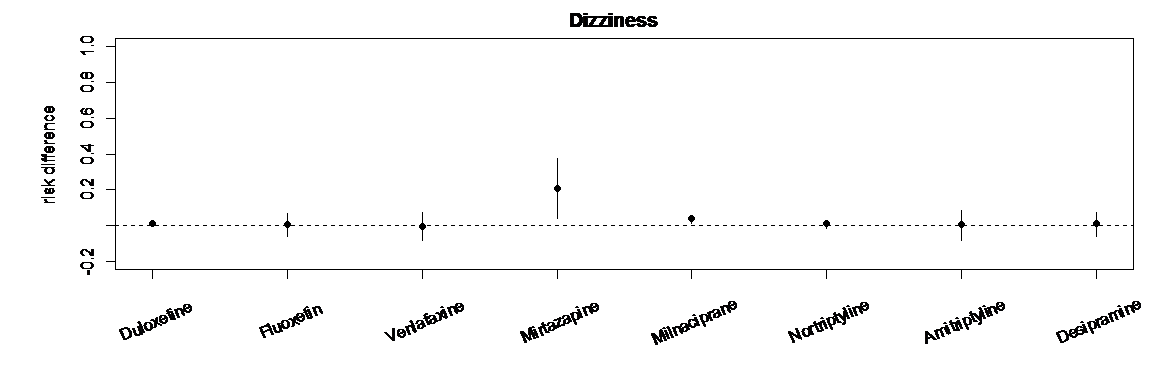


**Figure S-1 b:** Placebo effect- adjusted risk using risk differences for nausea


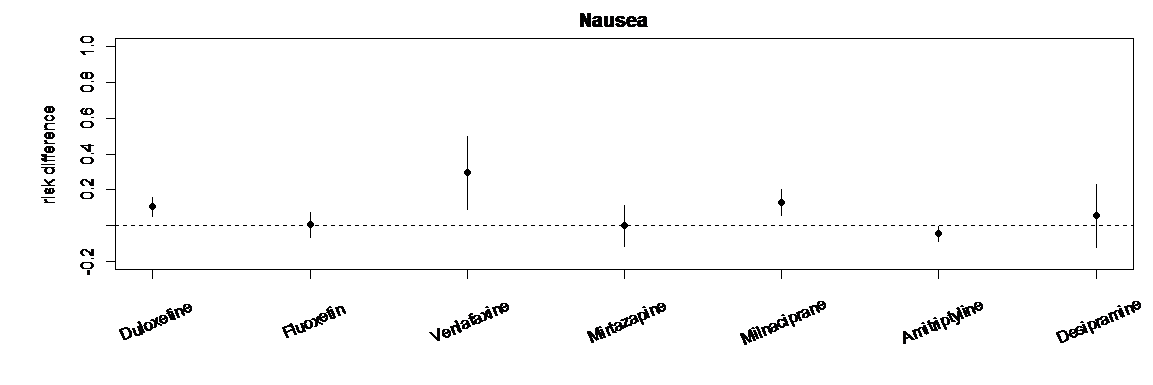


**Figure S-1 c:** Placebo effect- adjusted risk using risk differences for headache


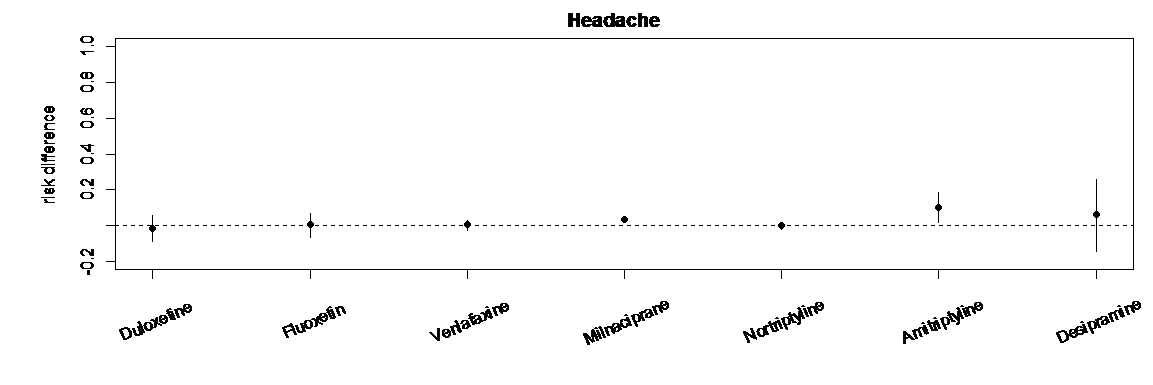


**Figure S-1 d:** Placebo effect- adjusted risk using risk differences for constipation


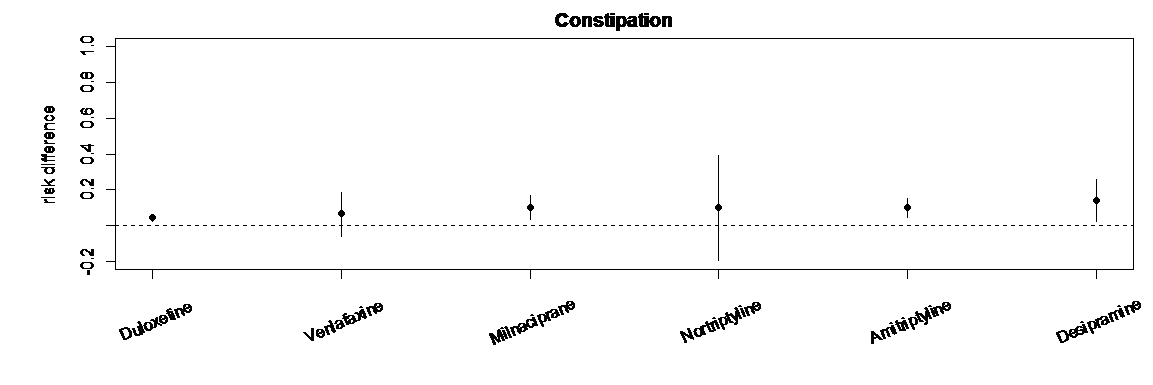


**Figure S-1 e:** Placebo effect- adjusted risk using risk differences for drowsiness


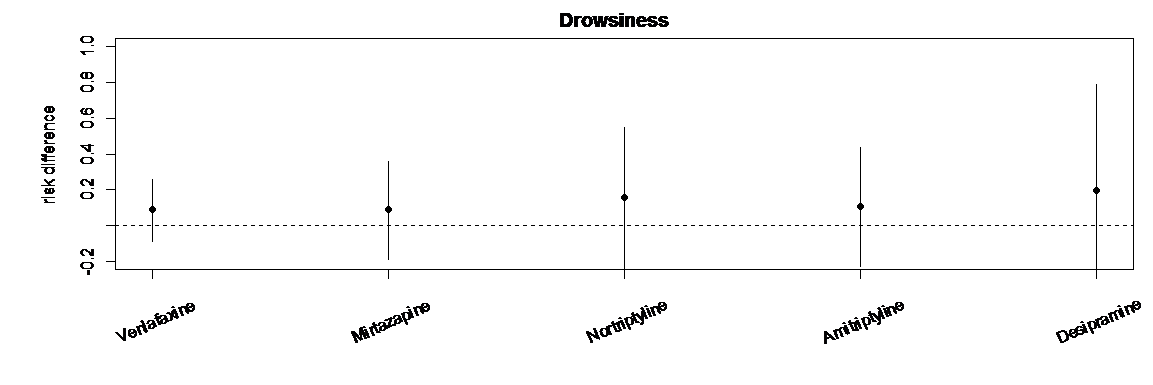


**Figure S-1 f:** Placebo effect- adjusted risk using risk differences for sweating


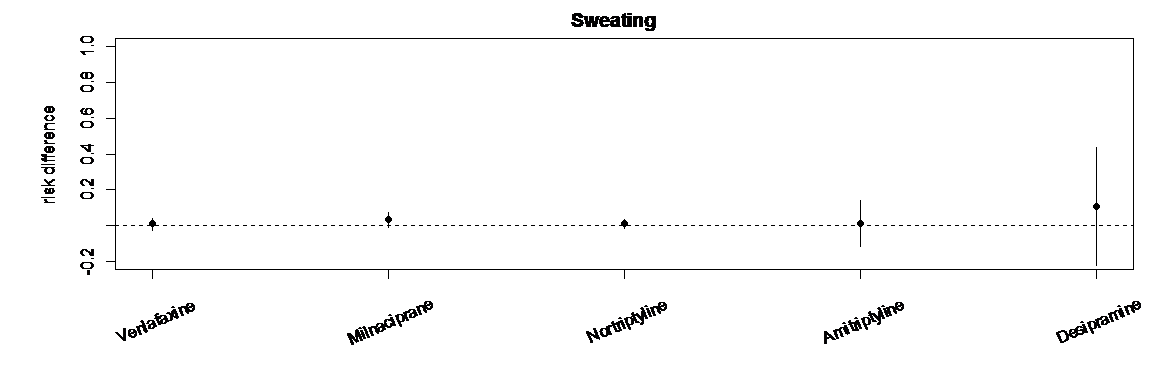


**Figure S-1 g:** Placebo effect- adjusted risk using risk differences for palpitations


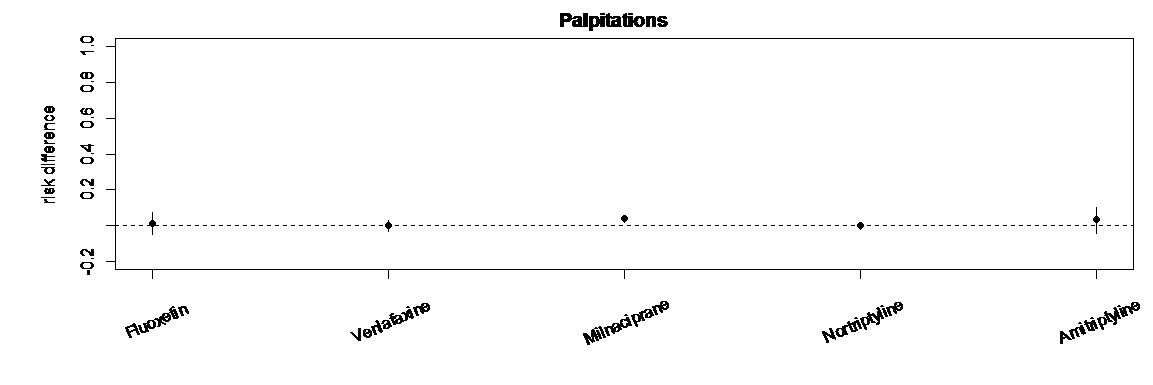

Supplement: Supplementary file 1 [file data_sheet_1.doc]
